# Bubble-templated synthesis of nanocatalyst Co/C as NADH oxidase mimic

**DOI:** 10.1093/nsr/nwab186

**Published:** 2021-10-11

**Authors:** Jinxing Chen, Xiliang Zheng, Jiaxin Zhang, Qian Ma, Zhiwei Zhao, Liang Huang, Weiwei Wu, Ying Wang, Jin Wang, Shaojun Dong

**Affiliations:** State Key Laboratory of Electroanalytical Chemistry, Changchun Institute of Applied Chemistry, Chinese Academy of Sciences, Changchun 130022, China; School of Applied Chemistry and Engineering, University of Science and Technology of China, Hefei 230026, China; State Key Laboratory of Electroanalytical Chemistry, Changchun Institute of Applied Chemistry, Chinese Academy of Sciences, Changchun 130022, China; State Key Laboratory of Electroanalytical Chemistry, Changchun Institute of Applied Chemistry, Chinese Academy of Sciences, Changchun 130022, China; School of Applied Chemistry and Engineering, University of Science and Technology of China, Hefei 230026, China; State Key Laboratory of Electroanalytical Chemistry, Changchun Institute of Applied Chemistry, Chinese Academy of Sciences, Changchun 130022, China; School of Applied Chemistry and Engineering, University of Science and Technology of China, Hefei 230026, China; State Key Laboratory of Electroanalytical Chemistry, Changchun Institute of Applied Chemistry, Chinese Academy of Sciences, Changchun 130022, China; School of Applied Chemistry and Engineering, University of Science and Technology of China, Hefei 230026, China; State Key Laboratory of Electroanalytical Chemistry, Changchun Institute of Applied Chemistry, Chinese Academy of Sciences, Changchun 130022, China; School of Applied Chemistry and Engineering, University of Science and Technology of China, Hefei 230026, China; State Key Laboratory of Electroanalytical Chemistry, Changchun Institute of Applied Chemistry, Chinese Academy of Sciences, Changchun 130022, China; School of Applied Chemistry and Engineering, University of Science and Technology of China, Hefei 230026, China; State Key Laboratory of Rare Earth Resource Utilization, Changchun Institute of Applied Chemistry, Chinese Academy of Sciences, Changchun 130022, China; Department of Chemistry and Physics, Stony Brook University, Stony Brook, NY 11794, USA; State Key Laboratory of Electroanalytical Chemistry, Changchun Institute of Applied Chemistry, Chinese Academy of Sciences, Changchun 130022, China; School of Applied Chemistry and Engineering, University of Science and Technology of China, Hefei 230026, China

**Keywords:** NADH oxidase, H_2_O_2_ production, oxidative phosphorylation

## Abstract

Designing highly active nanozymes for various enzymatic reactions remains a challenge in practical applications and fundamental research. In this work, by studying the catalytic functions of natural NADH oxidase (NOX), we devised and synthesized a porous carbon-supported cobalt catalyst (Co/C) to mimic NOX. The Co/C can catalyze dehydrogenation of NADH and transfers electrons to O_2_ to produce H_2_O_2_. Density functional theory calculations reveal that the Co/C can catalyze O_2_ reduction to H_2_O_2_ or H_2_O considerably. The Co/C can also mediate electron transfer from NADH to heme protein cytochrome c, thereby exhibiting cytochrome c reductase-like activity. The Co/C nanoparticles can deplete NADH in cancer cells, induce increase of the reactive oxygen species, lead to impairment of oxidative phosphorylation and decrease in mitochondrial membrane potential, and cause ATP production to be damaged. This ‘domino effect’ facilitates the cell to approach apoptosis.

## Teaser

A unique strategy with melted urea as solvent is developed to fabricate a porous carbon-supported cobalt catalyst to simulate the enzyme-catalyzed electron transfer process.

## INTRODUCTION

Following the first discovery of the intrinsic peroxidase-like activity of Fe_3_O_4_ and subsequent definition, nanozymes have flourished significantly [1–3]. Generally, nanozymes overcome the limitations of natural enzymes, such as easy deactivation, high costs, and being difficult to recycle, which makes nanozymes highly promising for substituting their natural counterparts [4–7]. However, after more than a decade of development, research into nanozymes has mainly been focused on simulation of peroxidase, oxidase, catalase, and superoxide dismutase [8–10]. To design more varieties of nanozymes, we simulated the active center of a natural enzyme to achieve specific catalytic properties. According to this bionic strategy, we prepared a single-atom FeN_5_ nanozyme to simulate natural cytochrome P450 oxidase and ZIF-8 to simulate carbonic anhydrase [11,12]. Although the biomimetic strategy has been successfully applied to the design of nanozymes, it faces two inevitable challenges. First, nanomaterials with a similar structure to the natural enzyme activity centers were very rare in the past reports. Second, *de novo* design and synthesis of new nanomaterials to simulate the subtly active centers of natural enzymes is very difficult.

To resolve these limitation issues, we propose a more flexible principle to design nanozymes by simulating the catalytic process of natural enzymes. For example, oxidation of NADH to H_2_O_2_ catalyzed by natural NADH oxidase (NOX) can be divided into two steps. In the first step, the flavin coenzyme of NOX catalyzes dehydrogenation of NADH to NAD^+^ and FADH_2_. In the second step, FADH_2_ transfers electrons and protons to O_2_ to generate superoxide and H_2_O_2_ [13]. Taken together, NOX has two catalytic features: dehydrogenation of NADH to obtain electrons and protons, then catalyzing the reduction of O_2_ to superoxide and H_2_O_2_ through the 1e^–^ and 2e^–^ path, respectively. Nanomaterials with these catalytic properties are highly promising to realize simulation of the functions of NOX.

For the catalytic function of dehydrogenation and hydrogenation, noble metal nanoparticles (NPs), such as Pt NPs and Pd NPs, exhibit prominent dehydrogenation and hydrogenation abilities to alkanes and alcohols [14]. To reduce costs, a large number of non-noble metal catalysts have been used to catalyze dehydrogenation and hydrogenation reactions, exhibiting considerable activity and selectivity. Among them, Co NPs are widely used in the dehydrogenation reactions and hydrogenation of N-heterocycles, the selective oxidation of alcohols, and Fischer–Tropsch syntheses [15]. Encapsulation of Co NPs with n-doped porous carbon is an effective approach for preventing aggregation and leaching of Co NPs, thus improving their activity and stability. Of note, Co-based catalysts have been found to selectively catalyze the reduction of O_2_ to H_2_O_2_ [16]. Therefore, Co NPs have catalytic functions similar to those of flavins, and thus they are highly promising in mimicking flavin enzymes.

The NOX mimics were chosen for research because NADH and NAD^+^ are coenzymes, which can be converted reversibly in various dehydrogenase and oxidase catalyzed reactions [17,18]. Therefore, the NOX mimics can be coupled to a dehydrogenase for continuous production of the green oxidant, H_2_O_2_, which is widely used in medical disinfection, wastewater treatment, industrial bleaching and chemical synthesis [19,20]. Furthermore, NADH is also a crucial electron source of the catalyzed electron transfer step in oxidative phosphorylation (OXPHOS) [21]. Although cancer cells are thought to synthesize ATP through glycolysis, the classic Warburg effect, recent studies have demonstrated that OXPHOS is also responsible for ATP production [22,23]. Therefore, respiratory complex I inhibitors have potential as anticancer drugs, which make the respiratory complex I dysfunctional in catalyzing oxidation of NADH and H^+^ transfers from the mitochondrial matrix to the mitochondrial intermembrane space. Blocking transport of H^+^ will lead to a decrease of mitochondrial membrane potential (Δψm), inhibit synthesis of ATP, and result in apoptosis of the cancer cells [24,25]. Inspired by this anticancer strategy, we speculate that NOX mimics can also be used to induce cancer cell apoptosis through consumption of NADH to competitively inhibit the function of respiratory complex I.

As a proof of concept, we synthesized multidimensional carbon-supported Co nanoparticles (Co/C) using an interesting and simple method. Uniform Co NPs embedded in carbon were obtained by pyrolysis of a homogeneous mixture of cobalt nitrate and imidazole. In the pyrolysis process, imidazole was polymerized and evaporated at the same time. The imidazole gas served as a template to obtain porous products. Taking the aerobic oxidation of NADH as a model reaction, we showed that Co/C can catalyze dehydrogenation of NADH and then transfer electrons and protons to O_2_ to generate H_2_O_2_, exhibiting a NADH oxidase-like activity. The selectivity of the O_2_ reduction to H_2_O_2_ was about 70%. Meanwhile, the Co/C was also found to have cytochrome *c* (Cyt *c*) reductase-like properties, in catalyzing transfer of electrons from NADH to Cyt *c*. After grinding Co/C into NPs, the obtained Co NPs can work in cancer cells to consume intracellular NADH. The NADH depletion leads to a series of consequences including OXPHOS impairment, Δψm decrease, ATP production inhibition, and, eventually, cancer cell death.

## RESULTS

### Synthesis and characterization of Co/C

**Figure 1.**
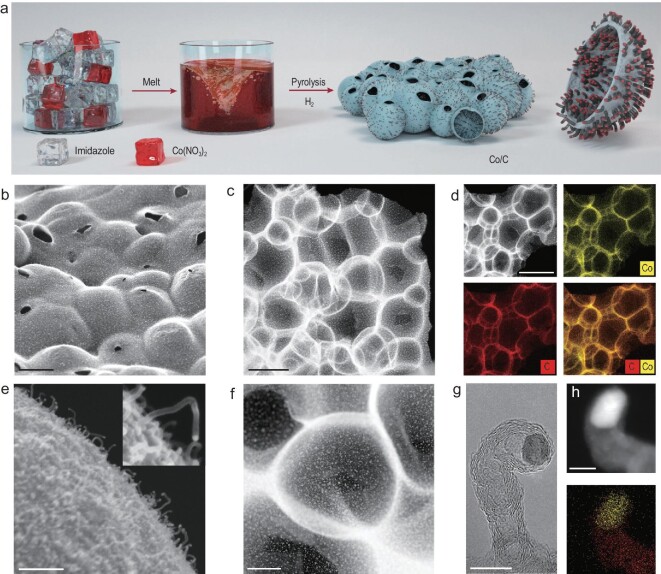
Synthesis and characterizations of Co/C. (a) Schematic illustration for the synthesis of Co/C. (b) SEM images of the Co/C. (c) HAADF-STEM images of the Co/C. (d) HAADF-STEM images along with the EDS maps of Co, and C. (e) SEM images of the CNT on Co/C surface. (f) HAADF-STEM images of the Co/C. (g) HRTEM images of the Co NPs embedded in the CNTs. (h) HAADF-STEM images of the Co NPs embedded in the CNTs, and the EDS maps of Co, and C. Scale bars: 4 μm (b), 2 μm (c), 2 μm (d), 200 nm (e), 1 μm (f), 10 nm (g) and 5 nm (h).

Figure 1a shows a schematic illustration of the synthesis of Co/C nanohybrids via the proposed simple two-step process. Briefly, imidazole and cobalt nitrate solids were heated at 120°C. The imidazole was melted rapidly into a liquid because its melting point is only 90°C. Then, the cobalt nitrate crystals were dissolved in liquid imidazole to form a uniform and transparent wine-red solution. The mixed solution was poured into a crucible and pyrolyzed in H_2_ atmosphere to obtain the hierarchical porous carbon-loaded Co NPs (Fig. S1).

Scanning electron microscopy (SEM) and transmission electron microscopy (TEM) images showed that the synthesized Co/C catalysts were shaped as three-dimensional porous carbon, consisting of closely packed hollow carbon spheres (Fig. 1b–d). Further magnification of the SEM images showed that the inner and outer surfaces of the hollow carbon spheres were covered with a large number of carbon nanotubes with outer diameters ranging from 10 to 20 nm (Figs 1e and S2). High-angle annular dark-field scanning transmission electron microscopy (HAADF-STEM) images showed that the Co NPs were uniformly distributed on the hollow carbon spheres (Fig. 1f). The layered structure with interlayer spacings of ∼3.6 Å was stacked in the multiwall of carbon nanotubes (CNTs), corresponding to the (002) planes of graphitic carbon (Fig. S3) [26]. High-resolution TEM images revealed crystalline Co NPs, which are seen to have specific lattice spacing of 2.04 Å and were embedded on top of the carbon nanotubes (Figs 1h and S3). Different magnifications of the HAADF-STEM images coupled with *in situ* energy-dispersive X-ray spectroscopy (EDS) elemental mapping confirmed the uniform distribution of the Co NPs on the entire carbon support (Figs 1d and S4).

The pyrolysis course of the imidazole-cobalt nitrate mixed solution into Co/C was explored by interval heating and calcination. Imidazole began to polymerize at about 250°C, and the products had specific porous morphology (Figs S5 and S6). Before heating up to 600°C, the products retained the morphology of porous carbon. No CNTs or Co NPs were observed in the SEM and TEM images (Fig. S7). However, Co elements were uniformly distributed in the pyrolysis products. When the temperature continued to rise to 600°C, Co NPs appeared, while no CNTs were formed (Fig. S8). At 700°C, the size of the Co NPs increased, and CNTs arose (Fig. S9). For the products calcined at 800°C and 900°C, there was no obvious change in the morphology of the products except that the size of the cobalt NPs increased with aggregation of the adjacent small-sized Co NPs (Figs S10 and S11). This is because the increase in temperature leads to a decrease in stability of the nanoparticles, therefore the nanoparticles will spontaneously aggregate to reduce the surface energy. The growth sequence of the Co NPs and CNTs and the specific position of the Co NPs wrapped on top of the CNTs suggested that the Co NPs were used as catalysts for the growth of the CNTs. In brief, the oriented formation process of Co/C undergoes simultaneous evaporation and pyrolysis of imidazole solutions, and the formation of Co NPs and the growth of CNTs were catalyzed by the Co NPs. In addition, as the carbon layer was decomposed with increase in temperature, the mass fraction of Co was gradually increased (Table S1).

### Mimicking NADH oxidase activity of Co/C

**Figure 2.**
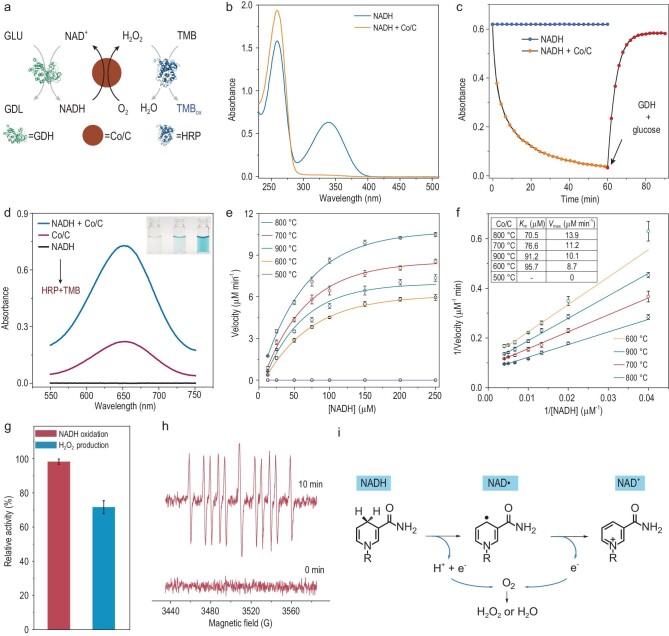
Mimicking NADH oxidase activity of Co/C. (a) Schematic representation of the tandem reactions catalyzed by GDH, Co/C and HRP. (b) The UV-Vis absorption spectra of NADH and the NADH + Co/C (40 μg mL^–1^) mixture after reacting for 60 minutes. (c) Time-dependent absorption change at 340 nm. GDH (0.2 U) and glucose (10 mM) were added to the reaction mixture at 60 minutes (Co/C was removed). (d) The UV-Vis absorption spectra of the different solutions reacting for 60 minutes after adding HRP and TMB. (e) Steady-state kinetic assay of the obtained Co/C at different temperatures. (f) Lineweaver-Burk plots obtained from Fig. 3e. (g) Co/C for the NADH oxidation reaction and the corresponding H_2_O_2_ selectivity. (h) Electron spin resonance (ESR) spectra of the spin adducts formed from CYPMPO in the reaction of NADH and Co/C. (i) Proposed mechanism of NADH oxidation and O_2_ reduction catalyzed by Co/C.

NADH/NAD^+^ are coenzymes for many kinds of oxidoreductases. NADH provides electrons and protons in oxidase-catalyzed reactions and is oxidized to NAD^+^, which participates in dehydrogenase-catalyzed reactions as an electron and proton acceptor and is reduced to NADH. Therefore, catalytic oxidation of NADH is of great significance for various cascade reactions (Fig. 2a) [24]. As NADH has a characteristic absorption peak at 340 nm, which disappears after oxidation, the oxidation of NADH can be determined by analyzing the absorption change. After mixing the NADH together with Co/C and incubating for 1 h, the characteristic absorption peak at 340 nm disappeared (Fig. 2b). The NADH oxidation rate was more remarkable in the O_2_ saturated solution, whereas it was slow in the N_2_ saturated solution (Fig. S18). These results demonstrate that Co/C possesses an NADH oxidase-like activity. In addition, Co/C can also catalyze the oxidation of NADPH (Fig. S19). It should be noted that NADH can be oxidized into biologically active cofactor NAD^+^, and it may also be oxidized to the inactive cofactor dimer (NAD)_2_. To verify the formation of NAD^+^, glucose and glucose dehydrogenases (GDHs) were added to the reaction system to reduce NAD^+^ to NADH. After addition of glucose and GDH, the characteristic absorption of NADH was recovered by ∼95% within 30 minutes. Such a high recovery demonstrates that NAD^+^ is the main oxidation product (Fig. 2c) [27].

In an oxidase-catalyzed reaction, O_2_ can be reduced to H_2_O by 4e^–^ pathways or to H_2_O_2_ by 2e^–^ pathways. The produced H_2_O_2_ can further be converted into free radicals, which can oxidize a variety of substrates and can be used for antibacterial pollutant degradation. Therefore, it is meaningful to catalyze the reduction of O_2_ to H_2_O_2_ with NADH as an electron donor. The H_2_O_2_ produced from the oxidation of NADH catalyzed by Co/C was detected using the colorimetric method. A characteristic absorption peak of TMBox at 652 nm can be detected by adding horseradish peroxidase (HRP) and 3,3^′^,5,5^′^-tetramethylbenzidine (TMB) into the mixture of NADH and Co/C (Fig. 2d). No absorption peaks were detected in the control group that contained only NADH. Interestingly, a weak absorption peak was detected in the control group that contained only Co/C. However, Co/C alone can hardly catalyze the oxidation of TMB (Fig. S20). This phenomenon indicates that H_2_O_2_ was automatically produced in the Co/C solution.

Typical Michaelis-Menten curves were obtained from the reaction rates in the NADH solutions with increasing concentration (Fig. 2e). The *K*_m_ values of the obtained samples at different calcination temperatures were lower than that of natural NOX (124 μM), indicating higher affinity to NADH [28]. The catalytic activity increased with increasing temperature because of the higher crystallinity of cobalt (Fig. 2f). When the temperature was further increased to 900°C, the size of the Co NPs increased, and the activity decreased. As there is no unified method to calculate the *k*_cat_ value of nanozymes, we measured the mass activity of Co/C. The mass activity of Co/C (1.2 U mg^–1^) is only 70 times lower than that of natural NOX (81.9 U mg^–1^). Considering that almost all the mass activities of nanozymes are lower than those of the corresponding natural enzymes (most are several orders of magnitude lower), the relative activity of Co/C is enough for practical catalytic reactions [9,29].

Generally, the oxygen reduction path was an incomplete 2e^–^ path to H_2_O_2_ or a 4e^–^ path to H_2_O [30]. We measured the ratio of the generated H_2_O_2_ to the consumed NADH, and found that the selectivity of the O_2_ reduction to H_2_O_2_ was about 68% in the NADH oxidation reaction (Fig. 2g). The Co/C catalysts can be separated for recycling uses, ∼90% of the initial activity was retained after five cycles of use, and the selectivity of H_2_O_2_ was maintained at ∼70% (Fig. S21). The catalytic activity of Co/C did not decrease obviously after 10 months’ storage in air (Fig. S22), but any decrease in activity may result from oxidation of some Co NPs in the buffer solution. As mentioned above, Co/C can react with O_2_ in buffer solutions according to the equation in Fig. S23. In the HEPES buffer solution, Co/C can produce small amounts of H_2_O_2_ within 10 minutes. The diffraction peak of XRD slightly changed after soaking for 2 hours, indicating that Co/C was relatively stable in HEPES. Nonetheless Co/C can rapidly produce a large amount of H_2_O_2_ (ca. 4.5 μmol mg^–1^) in PBS. The XRD patterns show that the Co/C can form cobalt phosphate in PBS. The etching effect of the phosphate on cobalt accelerates oxidation of the Co/C. In addition, the cobalt phosphate can be reduced to Co NPs by H_2_ (Fig. S24). Therefore, Co/C can serve as a reversible electron carrier offering a new way to generate H_2_O_2_*in situ*.

The electron spin-resonance spectroscopy with CYPMPO as a spin trapping agent was used to verify the dehydrogenation process of the cobalt-catalyzed NADH oxidation. The identical strong signal of the CYPMPO-NAD adduct was observed in the presence of NADH and Co/C (Fig. 2h) [31]. This result suggests that Co/C can dehydrogenate NADH to NAD• intermediate, which is similar to the natural NOX catalyzed process. Then O_2_ obtains electrons from NADH and is reduced to H_2_O or H_2_O_2_.

### Electrocatalytic ORR characterizations and theoretical calculations

**Figure 3.**
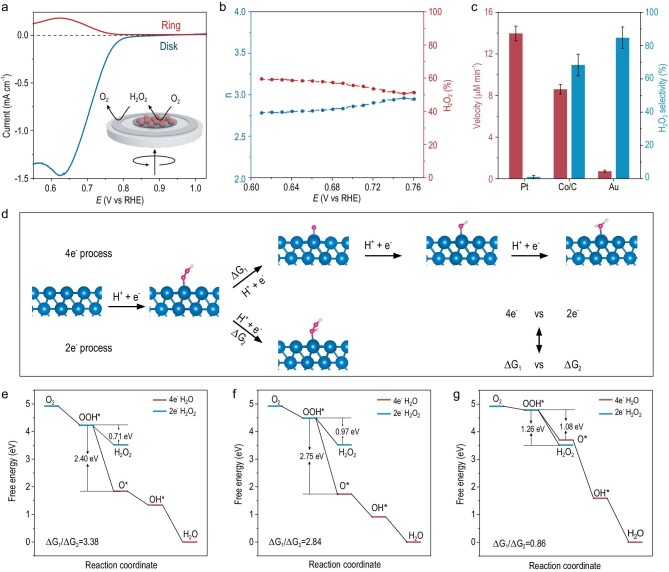
Electrocatalytic ORR characterizations and theoretical calculations. (a) RRDE measurement of the selective oxygen reduction of Co/C in the O_2_-saturated electrolyte (10 mM HEPES, 100 mM KCl, pH 7.4). insert: schematic diagram of the RRDE used to test the selectivity of the O_2_ reduction to H_2_O_2_. (b) Electron transfer number (n, left) and H_2_O_2_ selectivity (right) versus the potential for Co/C. (c) The aerobic oxidation rates of NADH and the selectivity of the O_2_ reduction to H_2_O_2_ catalyzed by Pt, Co/C, and Au (100 μM NADH, 20 μg/mL of catalysts, 10 mM HEPES, pH 7.4). (d) The diagram of four-electron and two-electron ORR pathways with adsorption of the oxygen-containing species on the Co (111) facets. The free energy diagram of Pt (e), Co/C (f) and Au (g) determined by the DFT studies.

The selectivity of the O_2_ reduction to H_2_O_2_ on the Co/C surface was also investigated at the electrode [32]. As shown in Fig. 3a, Co/C was loaded on the disk electrode of the rotating ring-disk electrode. In the negative scanning, the Co obtained electrons and then transferred them to O_2_ for the electrocatalytic oxygen reduction reaction (ORR). H_2_O_2_ was produced on the surface of the working electrode and diffused to the ring electrode with a fixed potential of 1.2 V. The selectivity of the reduction of O_2_ to H_2_O_2_ can be obtained by calculating the ratio of the H_2_O_2_ oxidation current at the ring electrode to the ORR current at the working electrode. The onset potential of the Co/C catalyst for ORR was 0.85 V. Correspondingly, the ring current increased and exhibited the same trend of change in the disk current, implying formation of H_2_O_2_. The electron transfer number of the Co/C catalyst for ORR was ca. 2.8, and the corresponding H_2_O_2_ selectivity was 65%, which is close to the selectivity of aerobic oxidation of NADH (Fig. 3b).

We also investigated the catalytic properties of Pt and Au for the NADH oxidation. As shown in Fig. 3c, the Pt-catalyzed NADH oxidation rate was the fastest, but no obvious H_2_O_2_ was detected. As for Au, the catalytic oxidation of NADH was slow, but the selectivity of H_2_O_2_ was high. This result is consistent with previous reports, that is, the Pt-catalyzed O_2_ reduction mainly generated H_2_O through a four-electron path, but the Au-catalyzed O_2_ reduction mainly generated H_2_O_2_ through a two-electron path [33,34]. Compared with Au, the oxidation rate of the NADH catalyzed by Co/C was faster, while the selectivity of the H_2_O_2_ catalyzed by Co/C was higher than that of Pt. Therefore, Co/C can be used to catalyze the conversion of NADH and can be coupled with the O_2_ reduction for the synthesis of H_2_O_2_.

Density functional theory (DFT) studies also supported this trend for selectivity of H_2_O_2_. The selectivity of H_2_O_2_ was evaluated by comparing the free energy of the watershed step of O_2_ reduction to H_2_O_2_ through the 2e^–^ pathway or to the H_2_O 4e^–^ pathway (Fig. 3d). The predicted free energy differences of the reductive H_2_O_2_ desorption step (*OOH + H^+^ + e^–^ → H_2_O_2_) and the hydrogenation OOH* to O* step (*OOH + H^+^ + e^–^ → O* + H_2_O) are shown in Fig. 3e–g, respectively. We evaluated the selectivity of H_2_O/H_2_O_2_ according to the ratio: ▵G_1_/▵G_2_. The results show that Pt with the largest ratio (3.38) has the greatest propensity to produce O* via the four-electron path. On the contrary, Au has the lowest ratio (0.86) and the Gibbs free energy of O* (▵G(O*)) is more positive than 3.52 eV (3.70 eV), it thermodynamically prefers formation of H_2_O_2_ relative to H_2_O via the two-electron path. Interestingly, the performance of Co/C with the medium ratio (2.84) is a compromise, that is to say, it can produce both H_2_O_2_ and H_2_O with comparable tendencies. The aforementioned DFT calculations are consistent with the experimental trends and, to some extent, give an underlying explanation of why Co/C is one of the best candidates of catalyst so far determined.

### Mimicking cytochrome c reductase activity of Co/C

**Figure 4.**
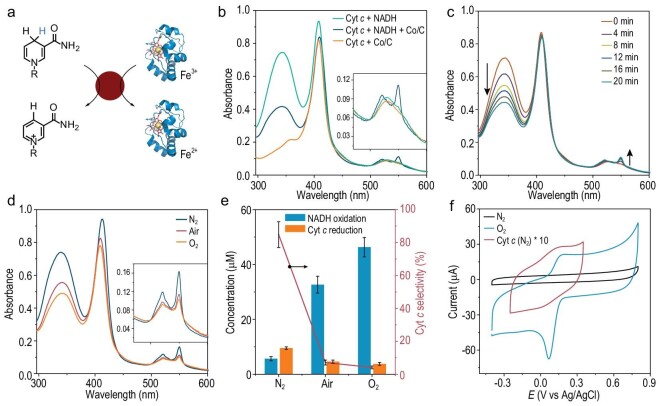
Mimicking cytochrome c reductase activity of Co/C. (a) Schematic representation of the catalyzed Cyt *c* reduction by Co/C. (b) The UV-Vis absorption spectra of the different solutions after reacting for 30 minutes. (c) Time-dependent absorption change of the reaction mixture containing Cyt *c*, NADH and Co/C. (d) The UV-Vis absorption spectra of the reaction mixture containing Cyt *c*, NADH and Co/C under different atmospheres. (e) NADH oxidation and Cyt *c* reduction under different atmospheres. (f) Cyclic voltammograms of Co/C in the N_2_- and O_2_-saturated solutions, and the cyclic voltammograms of Cyt *c* (0.4 mM) on the PEO-modified Au electrode (20 mM PBS, 50 mM NaClO_4_, pH 7.4).

Not only can Co/C transfer electrons from NADH to O_2_ but also to Cyt *c*, thus exhibiting Cyt *c* reductase-like properties (Fig. 4a). In the presence of NADH and Co/C, the absorption of the α band (550 nm) of ferric Cyt *c* increased, and the Soret band peak red-shifted from 409 to 412 nm, indicating/showing that the ferric Cyt *c* was converted to the ferrous form (Fig. 4b). The strength of the Soret band should have increased with reduction of Cyt *c*, but it decreased slightly, which may be a result of adsorption of Cyt *c* on the Co/C surface. NADH or Co/C alone did not induce spectral changes, demonstrating that Co/C can mediate electron transfer from NADH to Cyt *c*. The reduction of Cyt *c* was accompanied by oxidation of NADH, as can be seen from the increase in the absorption value at 550 nm and the decrease at 340 nm (Fig. 4c).

Considering that not only can Co/C transfer electrons from NADH to Cyt *c* but also to O_2_, we studied the selectivity of O_2_ and Cyt *c* as electron acceptors. In a N_2_ saturated solution, the absorption value at 340 nm slightly decreased, but the characteristic peak at 550 nm of the reduced Cyt *c* obviously increased, indicating that the electrons in NADH were mainly transferred to Cyt *c* (Fig. 4d). The Cyt *c* is the main electron acceptor, and because of the low concentration of Cyt *c*, the NADH consumption was very small. In contrast, in the O_2_ saturated solution, the absorption value at 340 nm rapidly decreased, while the characteristic peak of the reduced Cyt *c* slightly increased, indicating that the electrons in NADH were mainly transferred to O_2_. O_2_ competed with Cyt *c* and was more likely to be reduced. In the air saturated solution, the oxidation rate of NADH and the reduction rate of Cyt *c* were between those in the N_2_ saturated solution and O_2_ saturated solution (Fig. 4e). With the increase in the O_2_ concentration, the consumption rate of NADH was faster, and the reduction ratio of Cyt *c* decreased.

Cyclic voltammetry (CV) was performed to reveal the competition between O_2_ and Cyt *c*. At pH 7.4, the standard reduction potential of oxygen was 0.6 V (vs Ag/AgCl) (Fig. 4f). However, there was an overpotential of O_2_ on the catalyst surface, so it was necessary to determine the initial potential of the O_2_ reduction using a CV test. Under the N_2_ saturation condition, there was no reduction peak of Co/C in the CV curve. In the O_2_ saturated solution, an obvious reduction current appeared, corresponding to the reduction of oxygen, and the onset potential was 0.16 V. It was hard to measure the onset potential of Cyt *c* reduction as direct electron transfer of Cyt *c* on the electrode was difficult. Fortunately, the oxidation and reduction of Cyt *c* were reversible, so we could measure the onset reduction potential of Cyt *c* using a polyethylene oxide (PEO) Au electrode [35]. The reduction potential of Cyt *c* was about 0.05 V, lower than that of the O_2_ on the Co/C surface. As a result, compared with Cyt *c*, O_2_ is more likely to act as an electron acceptor in the dehydrogenation of NADH.

### Co/C catalyzes depletion of NADH in A549 lung cancer cells

**Figure 5.**
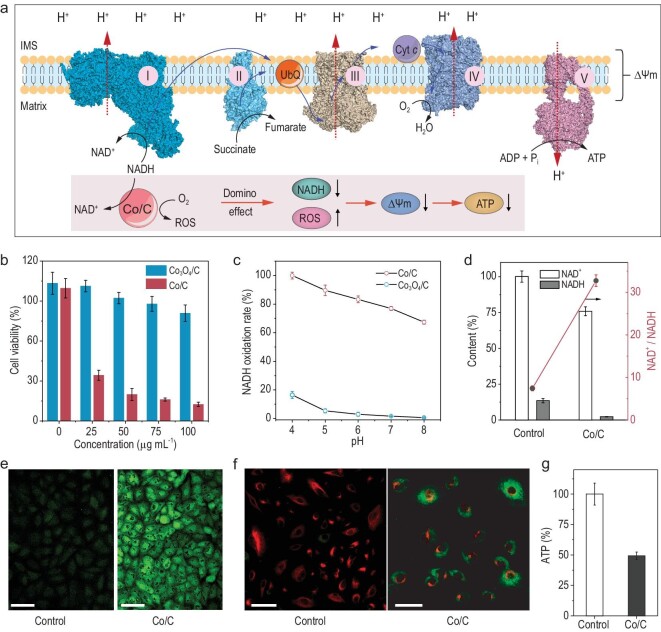
Co/C catalyzes depletion of NADH in A549 lung cancer cells. (a) Schematic representation of the ‘domino effect’ induced by Co/C nanoparticle-catalyzed NADH depletion. (b) A549 cell viability after treatment with Co/C or Co_3_O_4_ nanoparticles at different concentrations. (c) NOX-like activities of Co/C and Co_3_O_4_/C at different pH values. (d) Intracellular NAD^+^ and NADH in A549 cells treated with 50 μg mL^–1^ Co/C. (e) Intracellular ROS fluorescence images of A540 cells treated with 50 μg mL^–1^ Co/C. (f) Mitochondrial membrane potential (Δψm) change in A549 cells after treatment with 50 μg ml^–1^ Co/C. (g) Intracellular ATP in A549 cells treated with 50 μg mL^–1^ Co/C. Scale bars: 50 μm.

Figure 5a illustrates ATP production through oxidative phosphorylation (OXPHOS) in the cell’s mitochondrial inner membrane. The respiratory complex I catalyzes electron transfer from NADH to ubiquinone and release of 4H^+^ from the mitochondrial matrix to the intermembrane space (IMS). The electrons eventually transfer to O_2_ across complex III and complex IV accompanied by release of another 6H^+^ from the matrix to the IMS. In addition, succinate can also work as a part of the electron source to participate in the electron transfer chain (ETC) through complex II. The main function of the ETC is to increase the mitochondrial membrane potential (Δψm) by H^+^ transfer for ATP production through complex V. The crucial electron source, NADH, is produced through the tricarboxylic acid (TCA) cycle or by the malate-aspartate shuttle as well as glycerol-3-phosphate shuttle of cytosolic NADH [36]. Therefore, consumption of intracellular NADH by Co/C is promising to inhibit OXPHOS and ATP production.

The NOX-like activity of Co/C is retained by about 60% in human serum, which is a necessary condition for Co/C to work in complex physiological conditions (Fig. S25). The Co/C was ground into NPs to ensure ingestion by cells (Fig. S26). Confocal laser scanning microscope images show that the Co/C NPs accumulated within the cells (Fig. S27). Only 15% of A549 cell viability is retained after treatment with 100 μg mL^–1^ of Co/C NPs. For comparison, after treatment with 100 μg mL^–1^ of Co_3_O_4_/C NPs, the cell viability is 85%. Viability of the human normal liver cell (HL-7702) is 60% after treatment with 100 μg mL^–1^ of Co/C NPs. After being treated with 100 μg mL^–1^ of Co_3_O_4_/C NPs, cell viability can be retained at 100% (Fig. S28). The anticancer abilities of Co/C NPs and Co_3_O_4_/C NPs were highly relative to their NOX mimicking abilities. As shown in Fig. 5c, compared with Co_3_O_4_/C NPs, Co/C NPs have obvious superiority in their NOX-like activity. The NADH oxidation rates were more pronounced in acid solution, and this is advantageous in terms of consumption of NADH because of the acidic microenvironment in cancer cells. The catalytic activity of Co/C under neutral conditions is lower than that under acidic conditions, therefore, any damage caused by Co/C to normal cells is reduced.

We measured the intracellular NAD^+^ and NADH content change in A549 cells. The intracellular NAD^+^ concentration was reduced by ∼25% after incubation with Co/C NPs, while NADH concentration was dramatically reduced by up to 83%. The NAD^+^/NADH ratio was increased from 7 to 33, proving that Co/C NPs can catalyze NADH depletion in cells. In addition to participating in oxidative phosphorylation, NADH also provides the reducing power to maintain the redox balance in cells. The Co/C NPs not only consume the intracellular NADH but also produce a large amount of H_2_O_2_ in catalyzing the NADH oxidation reaction, which promotes a redox imbalance in cells [37]. Therefore, intracellular reactive oxygen species were evaluated using 2^′^,7^′^-dichlorodihydrofluorescein diacetate (DCFH-DA) as a fluorescent probe [38]. The typical green fluorescence was enhanced significantly in A549 cells after incubation with Co/C NPs, demonstrating that NADH consumption can produce ROS (Figs 5e and S25). Co/C can produce a large amount of H_2_O_2_ in water spontaneously, boosting accumulation of ROS.

The mitochondrial membrane potential (Δ*ψ*m) was evaluated using 5,5^′^,6,6^′^-tetrachloro-1,1^′^,3,3^′^-tetraethyl-imidacarbocyanine iodide (JC-1) as a fluorescent probe. The JC-1 keeps the form of monomer with green fluorescence (J-monomer) in mitochondria with low Δψm, while assembling into aggregates (J-aggregates) with red fluorescence in mitochondria with high Δψm [39,40]. As shown in Fig. 5f, JC-1 dye in control A549 cells exhibited red fluorescence because of the high Δψm. In the Co/C treated cells, the JC-1 manly exhibited green fluorescence, suggesting low Δψm. The decrease in Δψm implies that H^+^ cannot be transported from the mitochondrial matrix to the IMS because the NADH-dependent OXPHOS process was inhibited. Consequently, the ATP-producing ability was inhibited (Fig. 5g). These results demonstrate that the Co/C-catalyzed depletion of NADH can induce a ‘domino effect’ in facilitating the cell to approach apoptosis.

## CONCLUSION

In this work, by studying the catalytic properties of natural NOX, we designed and synthesized a Co/C catalyst to mimic the catalytic function of NOX. It was proven that Co/C can catalyze efficiently the oxidation of NADH to NAD^+^, accompanied by reduction of O_2_ to H_2_O_2_. Therefore, the NOX mimics can work with a dehydrogenase in a cascade reaction to continuously synthesize H_2_O_2_. In addition, the Co/C nanoparticles can work in cells to consume the intracellular NADH. NADH depletion can competitively inhibit the function of respiratory complex I and induce a ‘domino effect’, including increase in ROS, impairment of OXPHOS, decrease in Δψm, and decrease in ATP production. This combined effect causes apoptosis in cancer cells. The present work exemplifies that understanding the catalytic mechanism of natural enzymes contributes to design of highly efficient nanozymes, finding practical applications to replace their natural counterparts.

## Supplementary Material

nwab186_Supplemental_File
