# Supplementary material for: Bubble-templated synthesis of nanocatalyst Co/C as NADH oxidase mimic
Source: Natl Sci Rev. 2021 Oct 11;9(3):nwab186. doi: 10.1093/nsr/nwab186 (PMC8897313; doi:10.1093/nsr/nwab186)
Supplement: nwab186_Supplemental_File [file nwab186_supplemental_file.docx]

**Supplementary information**

**METHODS**

**Synthesis of Catalysts**

100 mmol of imidazole was heated to 120 °C to melt into a liquid. Then 2 mmol of Co(NO_3_)_2_ was added into the above imidazole solution. Co(NO_3_)_2_ dissolved rapidly in imidazole liquid to form wine red solution. After stirring for 5 minutes, the mixed solution was introduced into the alumina boat and calcined in hydrogen atmosphere at 800 °C to obtain Co/C (heating rate: 2 °C/min). The product obtained at 500 °C, 600 °C, 700 °C, and 900 °C were denoted as Co/C-500, Co/C-600, Co/C-700, and Co/C-900, respectively.

**Materials**

GDH (≥300 U·mg ^-1^) was purchased from Sigma-Aldrich. HRP (300 U·mg ^-1^) was received from Roche. 3,3’,5,5’-tetramethylbenzidine (TMB, 99%), Cobalt nitrate hexahydrate (Co(NO_3_)_2_ 6H_2_O, 99.99%), and imidazole (C_3_H_4_N_2_ , 99%) were acquired from Aladdin (Shanghai, China). Cell Counting Kit-8, NAD^+^/NADH Assay Kit, ATP Assay Kit 2′,7′-dichlorodihydrofluorescein diacetate (DCFH-DA), 5,5′,6,6′-tetrachloro-1,1′,3,3′-tetraethyl-imidacarbocyanine iodide (JC-1), and 1,1'-Dioctadecyl-3,3,3',3'-Tetramethylindocarbocyanine Perchlorate (DiI) were purchased from Beyotime (Shanghai, China). Human serum was obtained from the First Hospital of Jilin University. All chemicals were used as received without further purification. Ultrapure water was used throughout the experiments.

**Catalysts Characterizations**

TEM images were obtained with a Talos F200X microscope. SEM images were obtained with a ZEISS Sigma 300 field-emission microscope with an accelerating voltage of 5.0 kV. XRD analysis was carried out on a D8 Advance diffractometer with Cu Kα radiation (λ = 1.5406 Å). UV–vis absorption measurements were carried out on an Agilent Cary 60 UV-vis-near-infrared (NIR) spectrometer. Fluorescence spectra were collected from a Fluoromax-4 spectrofluorometer. ESR spectra were measured with a Bruker A300.

**NADH oxidase mimicking activity of Co/C**

In a typical experiment, NADH and Co/C were sequentially added into a vial containing 930 μL of 10 mM HEPES buffer (pH 7.4). The final concentration of NADH and Co/C were 0.1 mM and 20 μg mL^-1^, respectively. After 60 min incubation at room temperature, 20 μL HRP (0.1 mg mL^-1^) and 20 μL TMB (20 mM in DMSO:EtOH=1:9) were added into the above solution. UV-vis absorption measurements were performed within 30 s. For the NADH regeneration experiment, GDH (0.2 U) and glucose (10 mM) were added to the above mixture after the remove of Co/C.

**ORR tests**

The ORR performance of the Co/C was measured in HEPES buffer (10 mM, pH 7.4, 100 mM KCl as support electrolyte) at room temperature. A rotating ring-disk electrode (RRDE) modified with Co/C (980 μL of Co/C was mixed with 20 μL of 5wt% Nafion® solution) on the disk was served as the working electrode. Pt foil was served as the counter electrode and Ag/AgCl electrode was used as the reference electrode. Before electrochemical test, the electrolyte solutions were purged with O_2_ (or N_2_) for at least 30 mins. The LSV plots were recorded by applying proper potential ranges at the scan rate of 10 mV/s. The electron transfer number (n) and selectivity of the noble metal NPs toward H_2_O_2_ formation can be calculated according to the well known relation (eq 1 and eq 2):

$n=4\frac{\left| I_{disk} \right|}{\left| I_{disk} \right|+I_{ring}/N}$ (eq 1)

$H_{2}O_{2}\%=200\frac{I_{ring}}{N\left| I_{disk} \right|+I_{ring}}$ (eq 2)

where *I*_ring_ is the ring current, *I*_disk_ is the disk current and N is the collection efficiency (0.26).

**Cytochrome c reductase mimicking activity of Co/C**

In a typical experiment, NADH, Cyt c and Co/C were added sequentially into a vial containing 860 μL of 10 mM HEPES buffer (pH 7.4). The final concentration of NADH, Cyt c and Co/C were 100μM, 10 μM and 10 μg mL ^-1^ respectively. After 5 min incubation at room temperature, the supernatant was taken out for UV–vis absorption measurements.

**DFT calculations**

The Vienna ab initio simulation package (VASP, version 5.2) was used for the DFT calculations in this study. The projector augmented wave (PAW) method was applied to approximately describe the ionic cores, the Perdew, Burke and Ernzerh of functional (PBE) was utilized to evaluate the exchange and correlation energies. We have chosen 450eV as the cutoff energy for electronic wave functions, the parameter σ = 0.05 eV for the Gaussian electron smearing method. For the geometric optimization, the forces 0.01 eV Å^−1^ and the energy 10^−5^ eV were used as the convergence criterion. Spin-polarization was included to obtain the energies of the Co (111) adsorption systems. The lattice parameters for the nanoparticle Pt, Co, Au are determined by minimizing the total energy of the unit cell according to a conjugated gradient algorithm to relax the ions for bulk optimization. For the pure Pt, Co, Au, a 7 × 7 ×7 Monkhorst–Pack k-point grid is utilized to sample the Brillouin zone. For the corresponding periodically repeated slab models, the vacuum layer was set at 15 Å. Dipole correction was also included in this study. A 3 × 3 × 1 Monkhorst–Pack k-point sampling was used for surface structural relaxation and total energy calculation.

There are two elementary reaction steps in 2e- ORR pathway:

*+O_2_ + H^+^ + e^-^ → *OOH (1)

*OOH + H^+^ + e^-^ → H_2_O_2_+* （2）

The free energy difference of the reductive H_2_O_2_ desorption step is denoted by △G_2_:

*OOH + H^+^ + e^+^ → H_2_O_2_ (3)

The free energy difference of the hydrogenation OOH* to O* is described by △G_1_:

OOH* + H^+^ + e^+^ → O* + H_2_O (4)

In this study, we have quantified the selectivity of H_2_O/H_2_O_2_ by defining the selectivity ratio=△G_1_/△G_2_. Meanwhile, the equilibrium potential of 4e^-^ path (1.23 V vs. RHE) and 2e^-^ path (0.70 V vs. RHE) is used to determine the free energies of O_2_ and H_2_O_2_ molecules.

**Cell viability assay**

A549 cells were seeded at 10^4^ cells/well in 96-well culture plates with 200 μL DMEM per well. After 24 h incubation, A549 cells were treated with different concentrations of Co/C for 24 h. Then the cell viability was determined using a Cell Counting Kit-8 (CCK-8) (beyotime) according to the manufacturer’s instructions.

**Cellular Uptake**

A549 cells were seeded at 5×10^4^ cells/well in confocal dishes with a glass bottom (20 mm in diameter) with 2 mL DMEM. After 24 h of cell attachment, the DMEM was replaced with fresh DMEM containing Co/C (100 μg mL^-1^) and incubated for another 6 h, the cells were washed and stained with DiI working solution at 37 °C for 20 min and imaged by fluorescence microscope.

**Intracellular NADH and NAD^+^ content**

A549 cells were seeded at 10^4^ cells/well in 96-well culture plates with 200 μL DMEM per well. After 24 h of cell attachment, the DMEM was replaced with fresh DMEM containing Co/C (100 μg mL^-1^) and incubated for another 12h. Then the intracellular NAD^+^ and NADH contents were determined using NAD^+^/NADH Assay Kit with WST-8 (beyotime).

**Intracellular ATP level**

A549 cells were seeded at 10^4^ cells/well in 96-well culture plates with 200 μL DMEM per well. After 24 h of cell attachment, the DMEM was replaced with fresh DMEM containing Co/C (100 μg mL^-1^) and incubated for another 12h. Then the intracellular ATP content was determined using an ATP Assay Kit (beyotime).

**Intracellular ROS Detection**

A549 cells were seeded at 5×10^4^ cells/well in confocal dishes with a glass bottom (20 mm in diamter) with 2 mL DMEM. After 24 h of cell attachment, the DMEM was replaced with fresh DMEM containing Co/C (100 μg mL^-1^) and incubated for another 6 h, the cells were washed and stained with DCFH-DA working solution at 37 °C for 20 min and imaged by fluorescence microscope.

**Mitochondrial membrane potentials**

A549 cells were seeded at 5×10^4^ cells/well in confocal dishes with a glass bottom (20 mm in diameter) with 2 mL DMEM. After 24 h of cell attachment, the DMEM was replaced with fresh DMEM containing Co/C (50 μg mL^-1^) and incubated for another 12 h, the cells were washed and stained with JC-1 working solution at 37 °C for 20 min and imaged by fluorescence microscope.


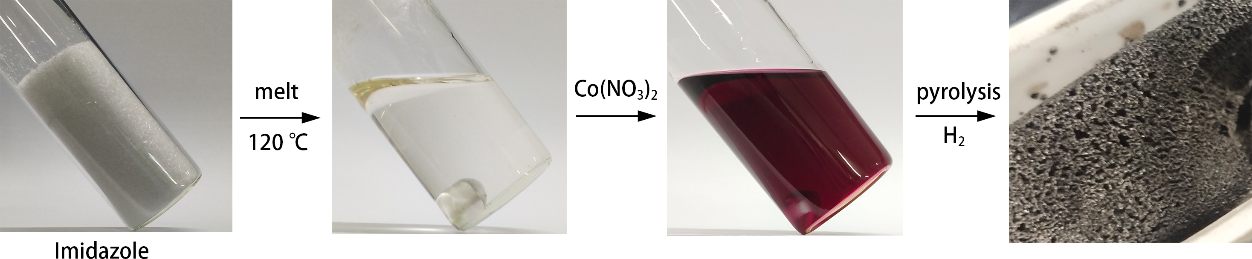


Figure s1. The steps of synthesizing Co/C.


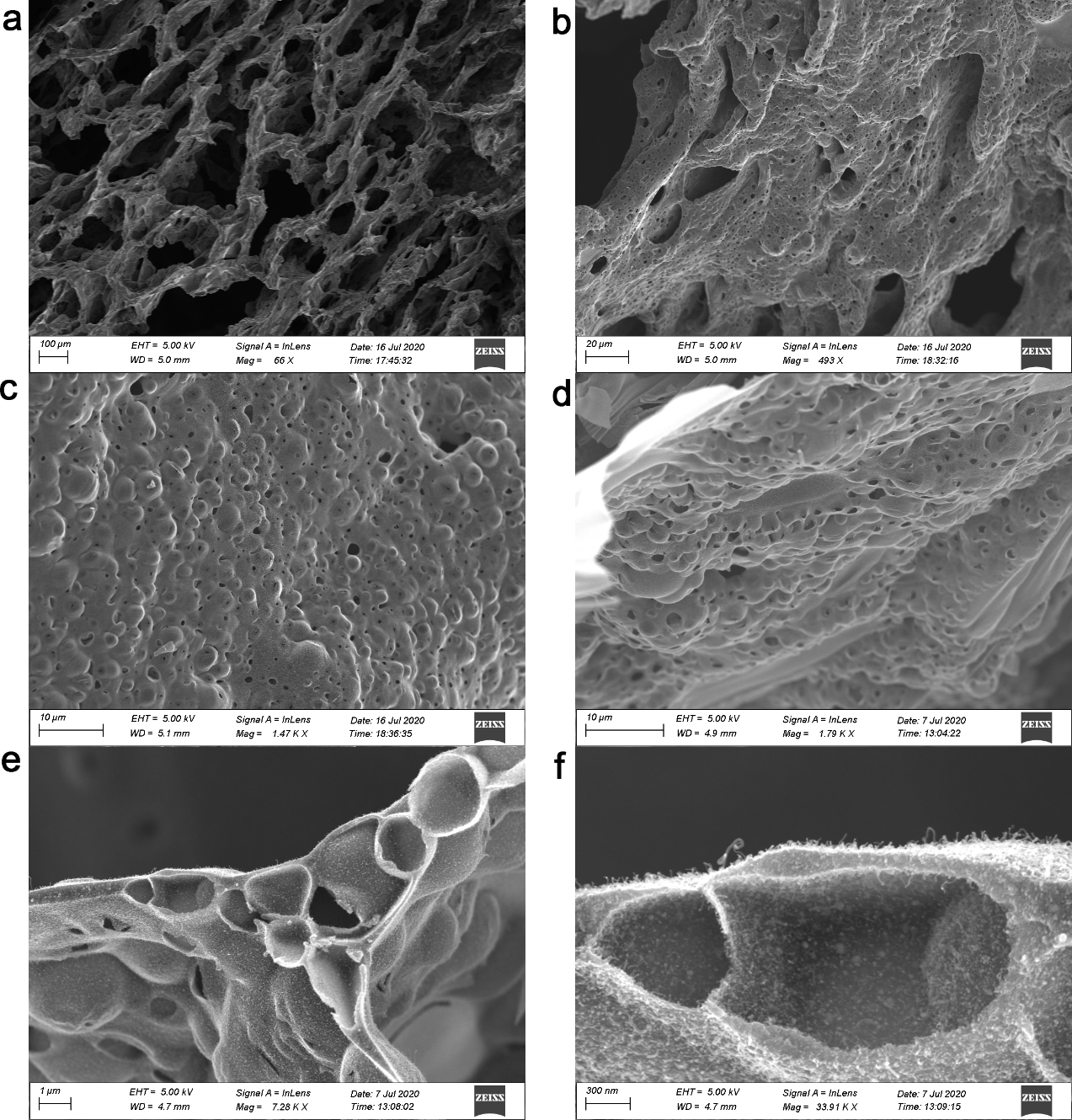


Figure s2. SEM image of the Co/C with different magnifications.


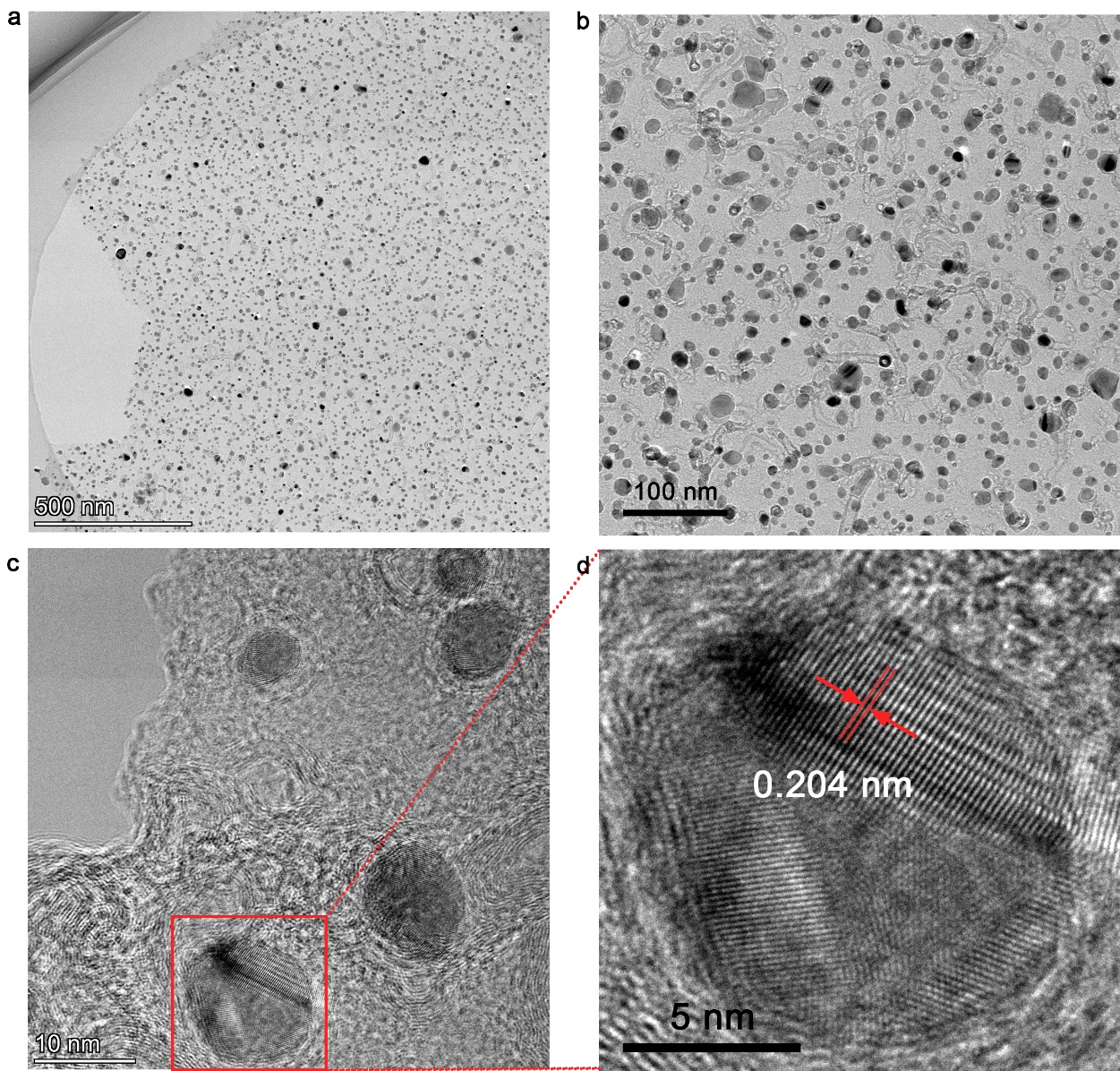


Figure s3. TEM image of the Co/C with different magnifications.


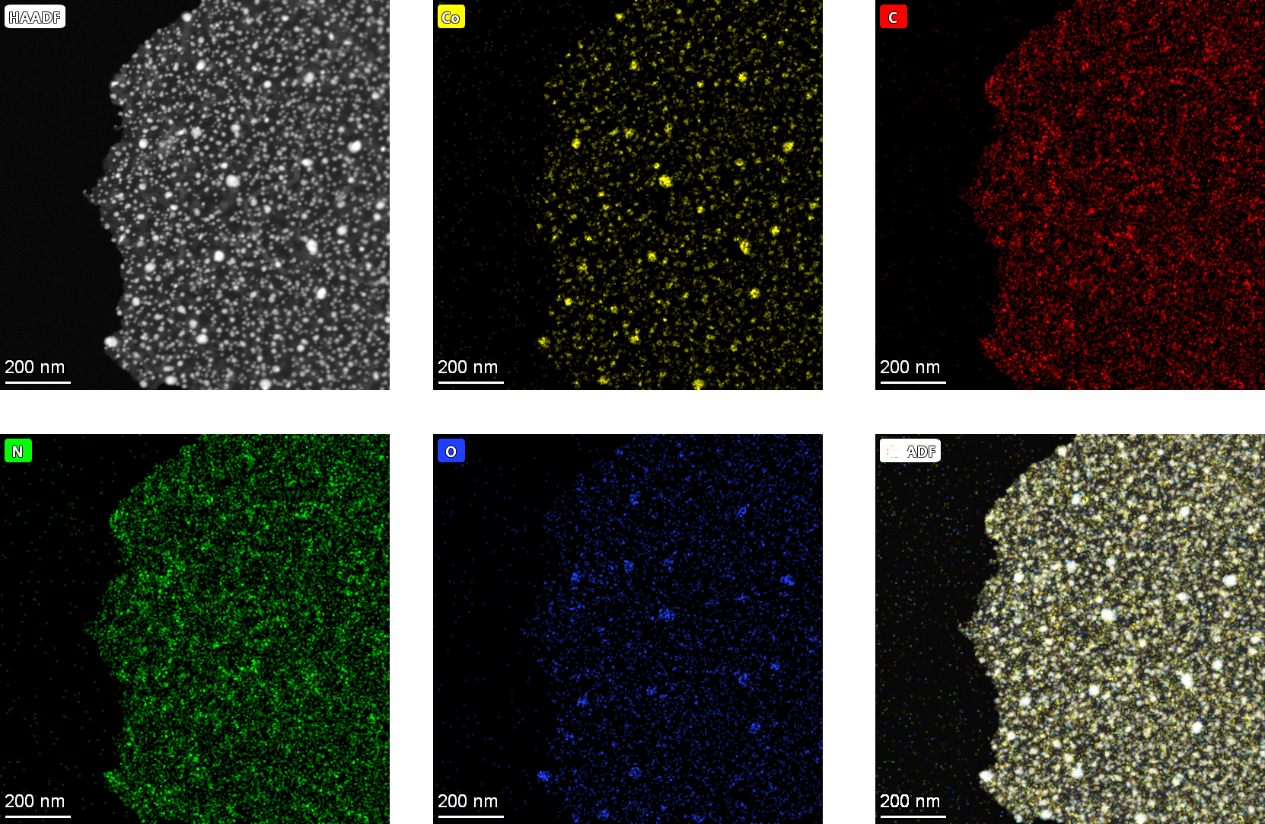


Figure s4. HAADF-STEM image of the Co/C along with the EDS maps of Co, C, N, and O.


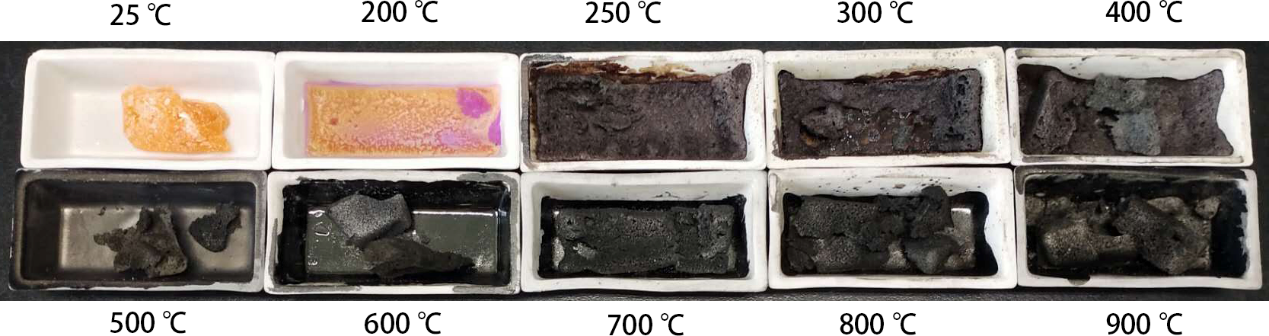


Figure s5. Photos of the mixture of imidazole and cobalt nitrate after pyrolysis at different temperatures.


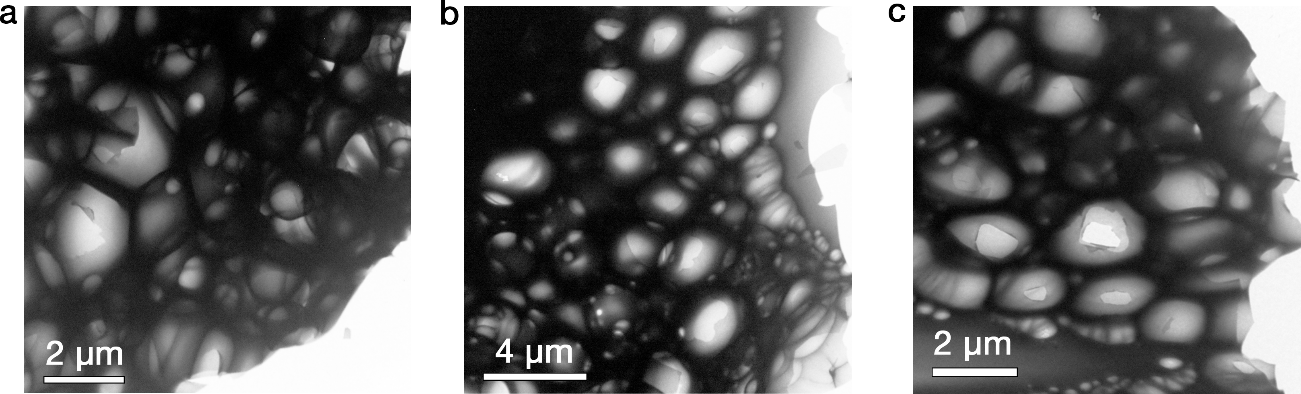


Figure s6. TEM images of the mixture of imidazole and cobalt nitrate after pyrolysis at 250 °C, 300 °C, and 400 °C temperatures.


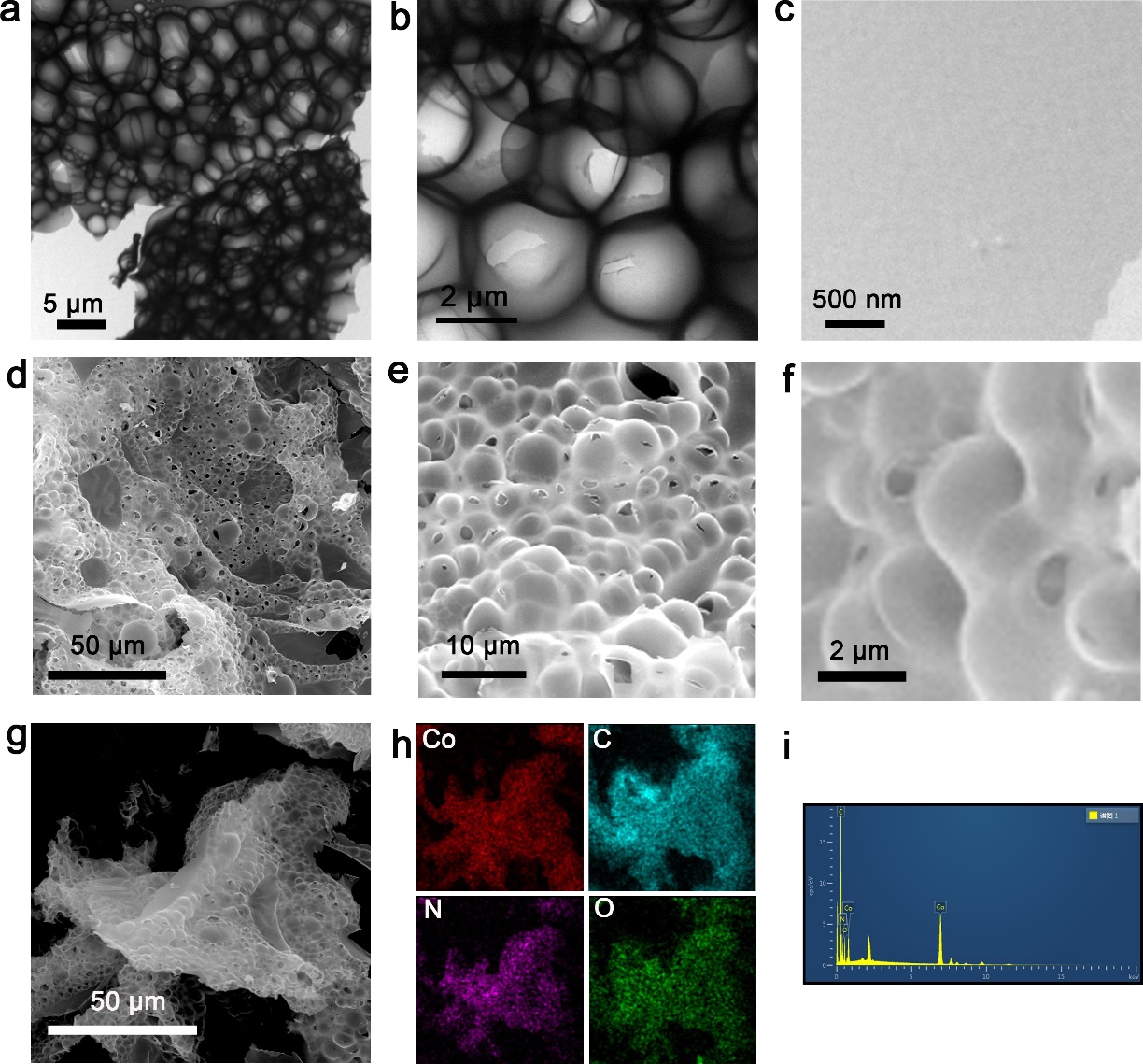


Figure s7. TEM images (a-c) and SEM images (d-f) of Co/C-500. SEM images along with the EDS maps of Co, C, N, and O. (g, h). The energy spectrum of Co/C-500.


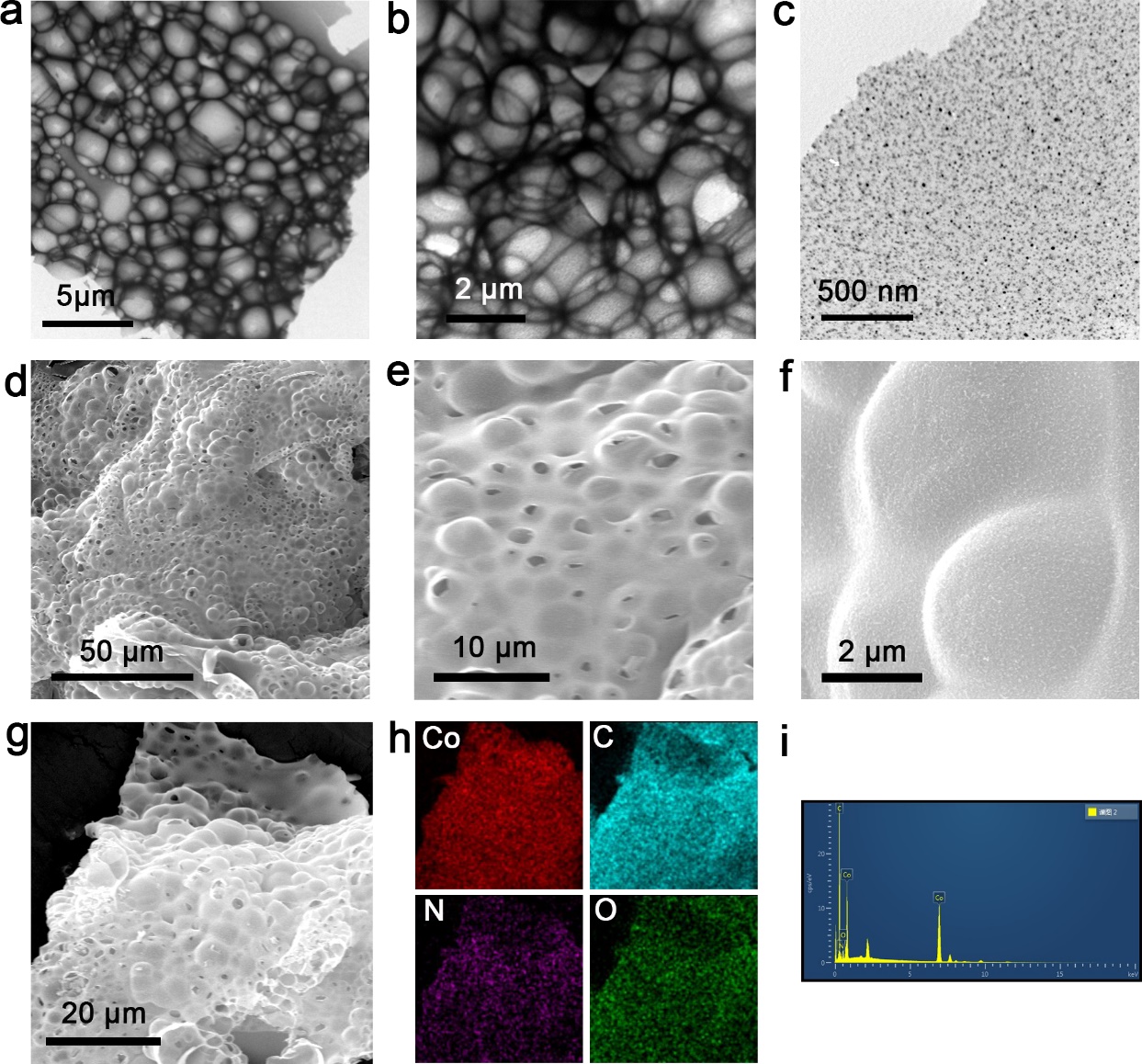


Figure s8. TEM images (a-c) and SEM images (d-f) of Co/C-600. SEM images along with the EDS maps of Co, C, N, and O. (g, h). The energy spectrum of Co/C-600.


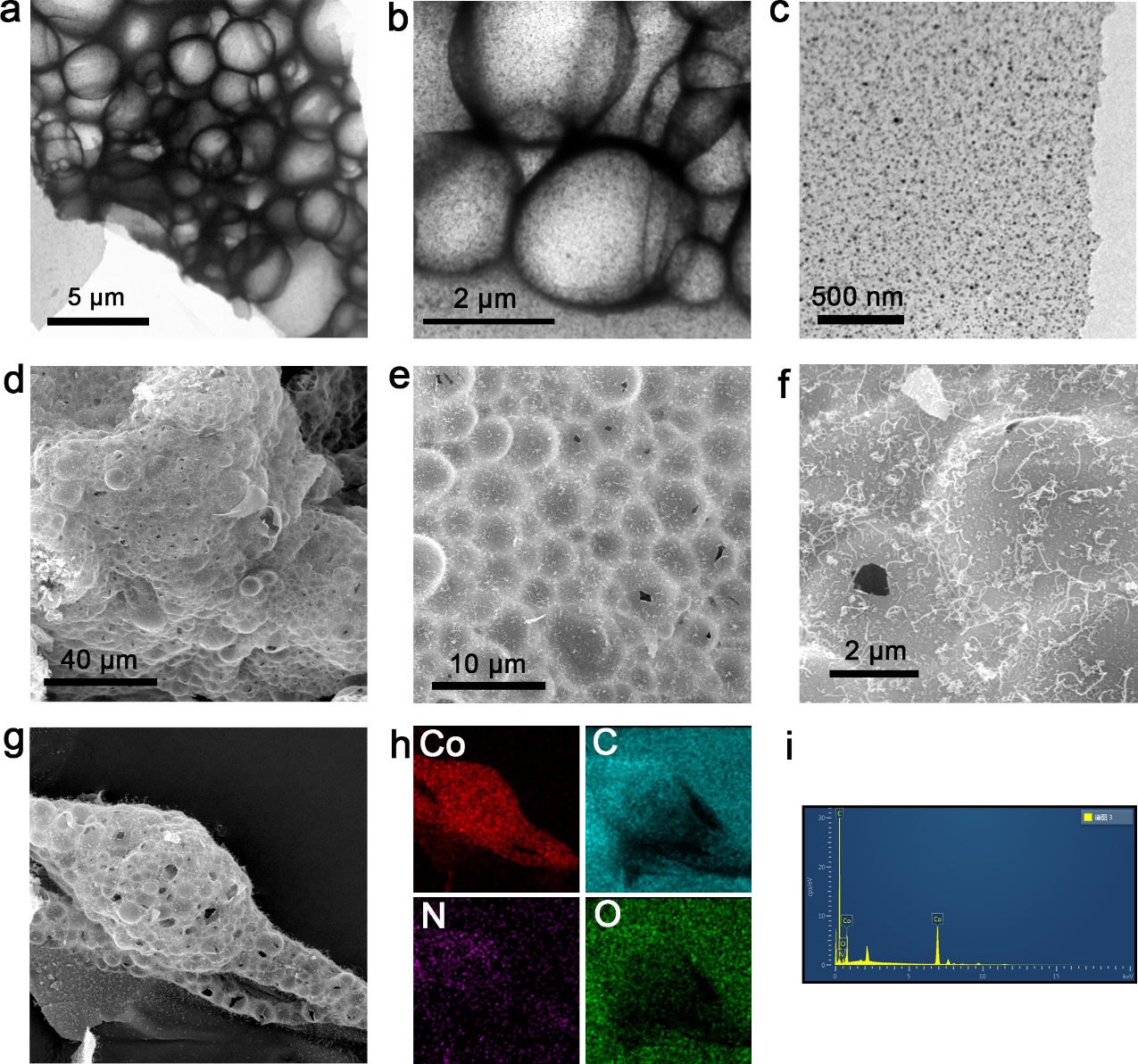


Figure s9. TEM images (a-c) and SEM images (d-f) of Co/C-700. SEM images along with the EDS maps of Co, C, N, and O. (g, h). The energy spectrum of Co/C-700.


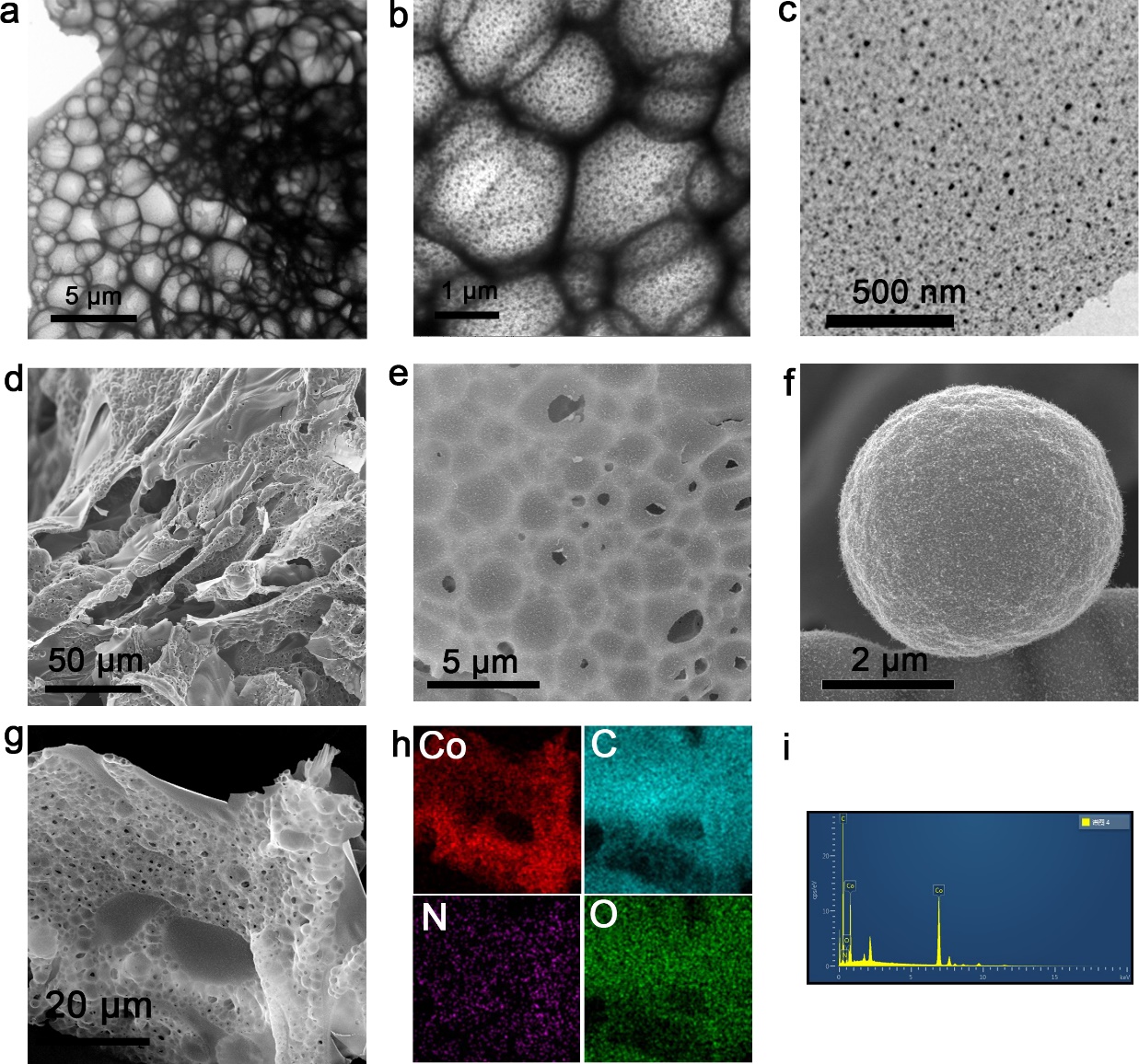


Figure s10. TEM images (a-c) and SEM images (d-f) of Co/C-800. SEM images along with the EDS maps of Co, C, N, and O. (g, h). The energy spectrum of Co/C-800.


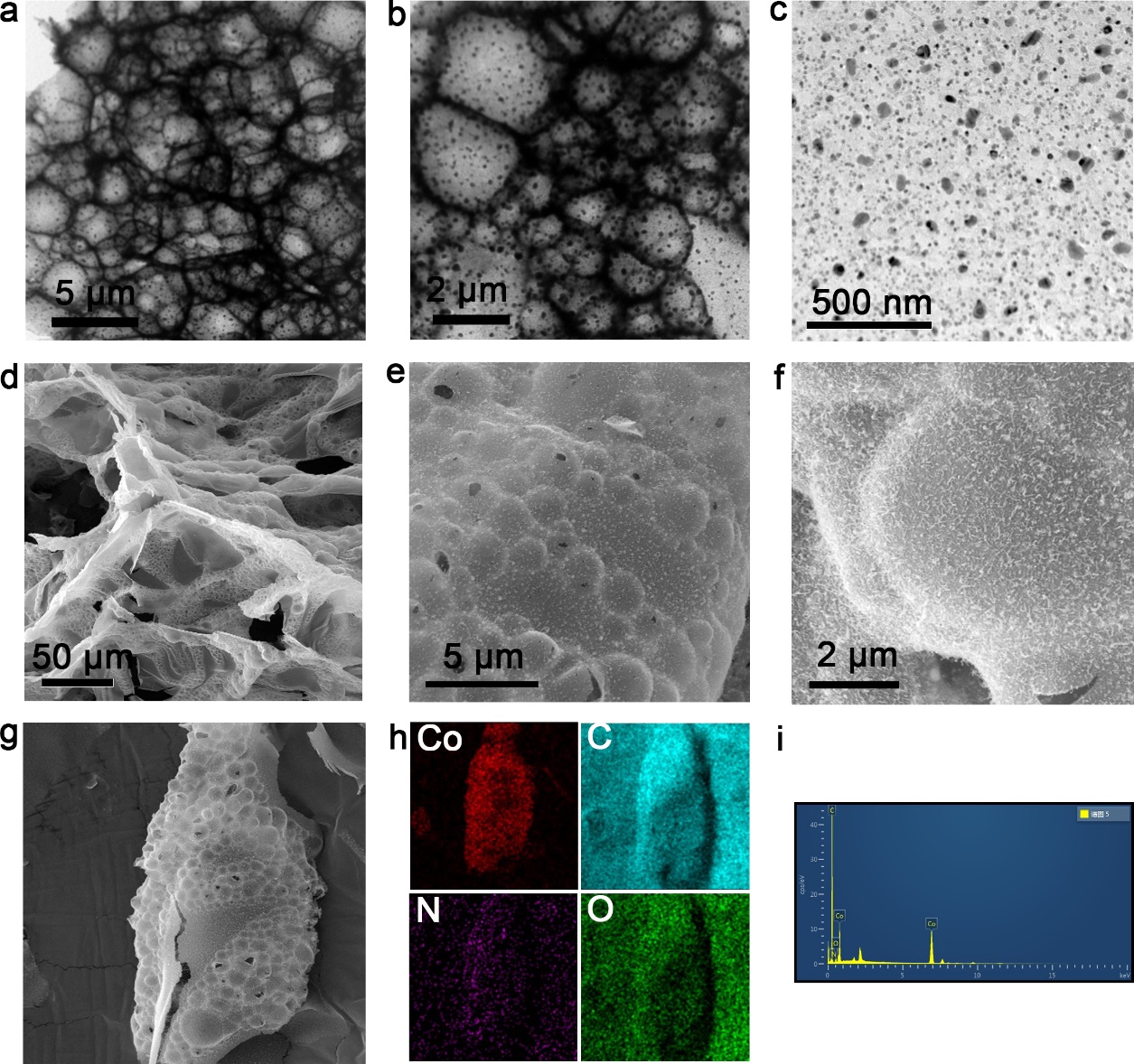


Figure s11. TEM images (a-c) and SEM images (d-f) of Co/C-900. SEM images along with the EDS maps of Co, C, N, and O. (g, h). The energy spectrum of Co/C-900.


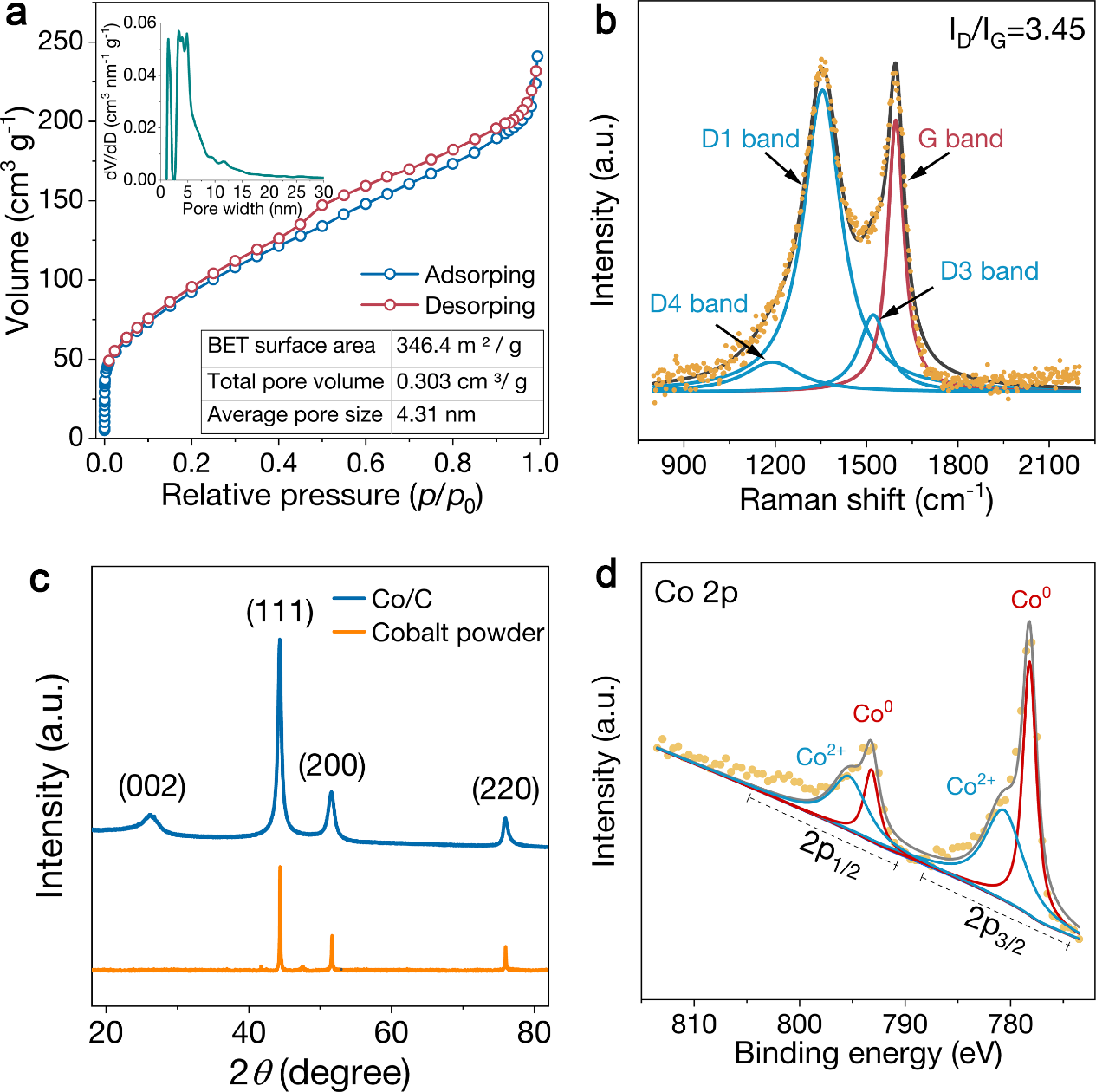


Figure s12. (a) N_2_ adsorption−desorption isotherm and pore size distribution (inset) using the DFT method of Co/C. (b) Raman spectrum of Co/C. (c) XRD pattern of Co/C. (d) High-resolution Co 2p X-ray photoelectron spectroscopy (XPS) spectrum of Co/C.

Brunauer−Emmett−Teller (BET) analyses were performed to determine the porous characteristics of the Co/C. The N_2_ adsorption/desorption curves of Co/C displayed typical type-IV isotherms with a hysteresis loop, implying a mesoporous structure. The specific surface area of Co/C was 346.4 m^2^ g^−1^, and the pore size distribution was centered at 4.31 nm (Fig. S12). The XRD patterns of Co/C showed the same three diffraction peaks (44.3°, 51.6°, and 76.0°) of the Co power, which corresponds to the (111), (200), and (220) planes of the metallic Co with the cubic structure, respectively (Fig. S12). The peak in the high-resolution X-ray photoelectron spectroscopy (XPS) spectra of Co/C at the binding energy of 780.9 eV consistent with the CoOx species, and the major peak at 778.2 eV could be attributed to Co^0^ (Fig. S12).


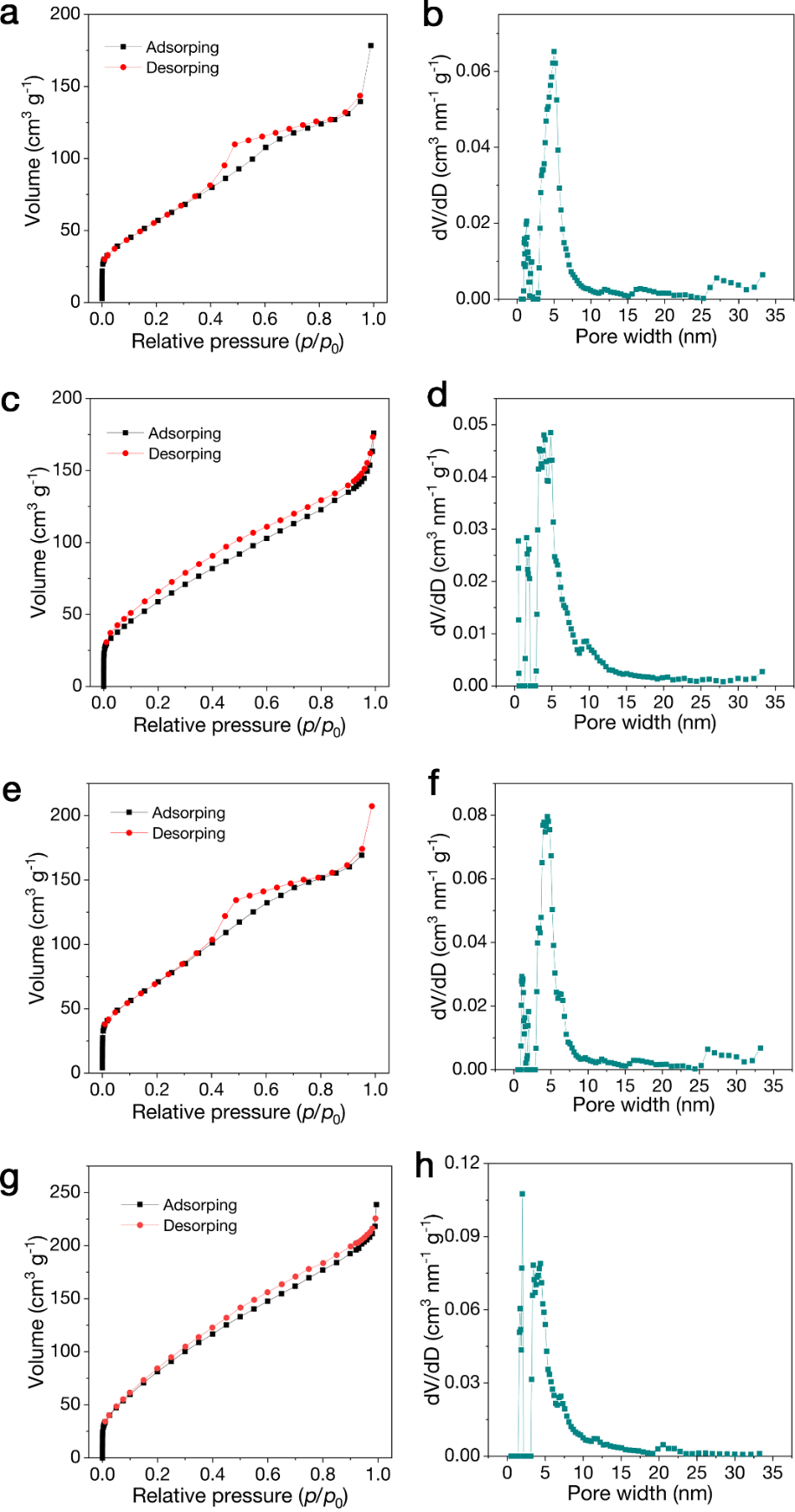


Figure s13. N_2_ adsorption−desorption isotherm and pore size distribution using the DFT method of Co/C-500 (a,b), Co/C-600 (c, d), Co/C-700 (e, f), Co/C-900 (g, h).

The BET surface area of the samples displayed a volcano-type variation versus the calcination temperature (Table S2, fig. S13).


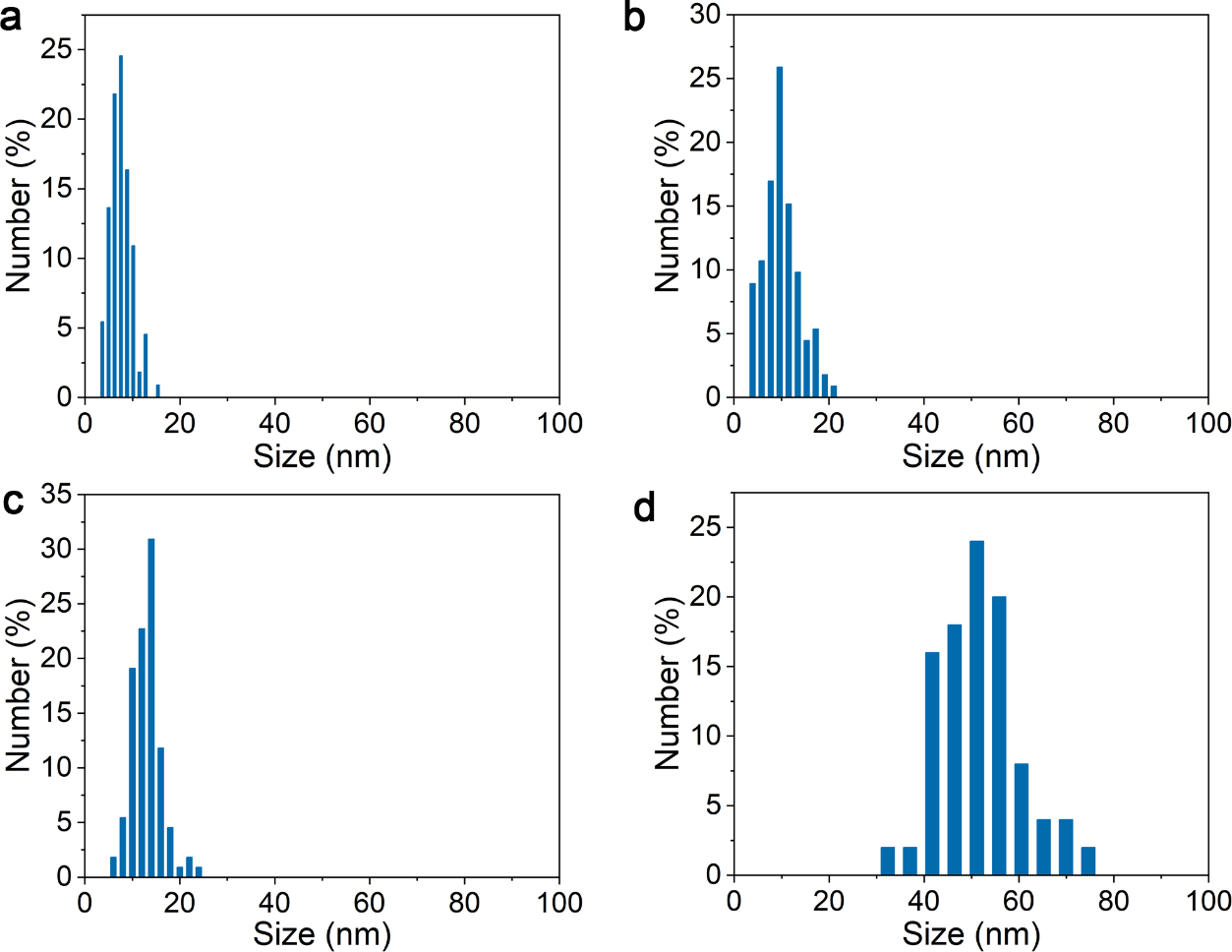


Figure s14. The size distribution of Co NPs in Co/C-600 (a), Co/C-700 (b), Co/C-800 (c), and Co/C-900 (d).

The increase in temperature promoted the pyrolysis of the carbon base and the formation of CNTs, resulting in an increase in the surface area. However, the decrease in the surface area of the samples calcined at 900°C was assumed to be due to the growth and aggregation of the Co NPs (Fig. S14).


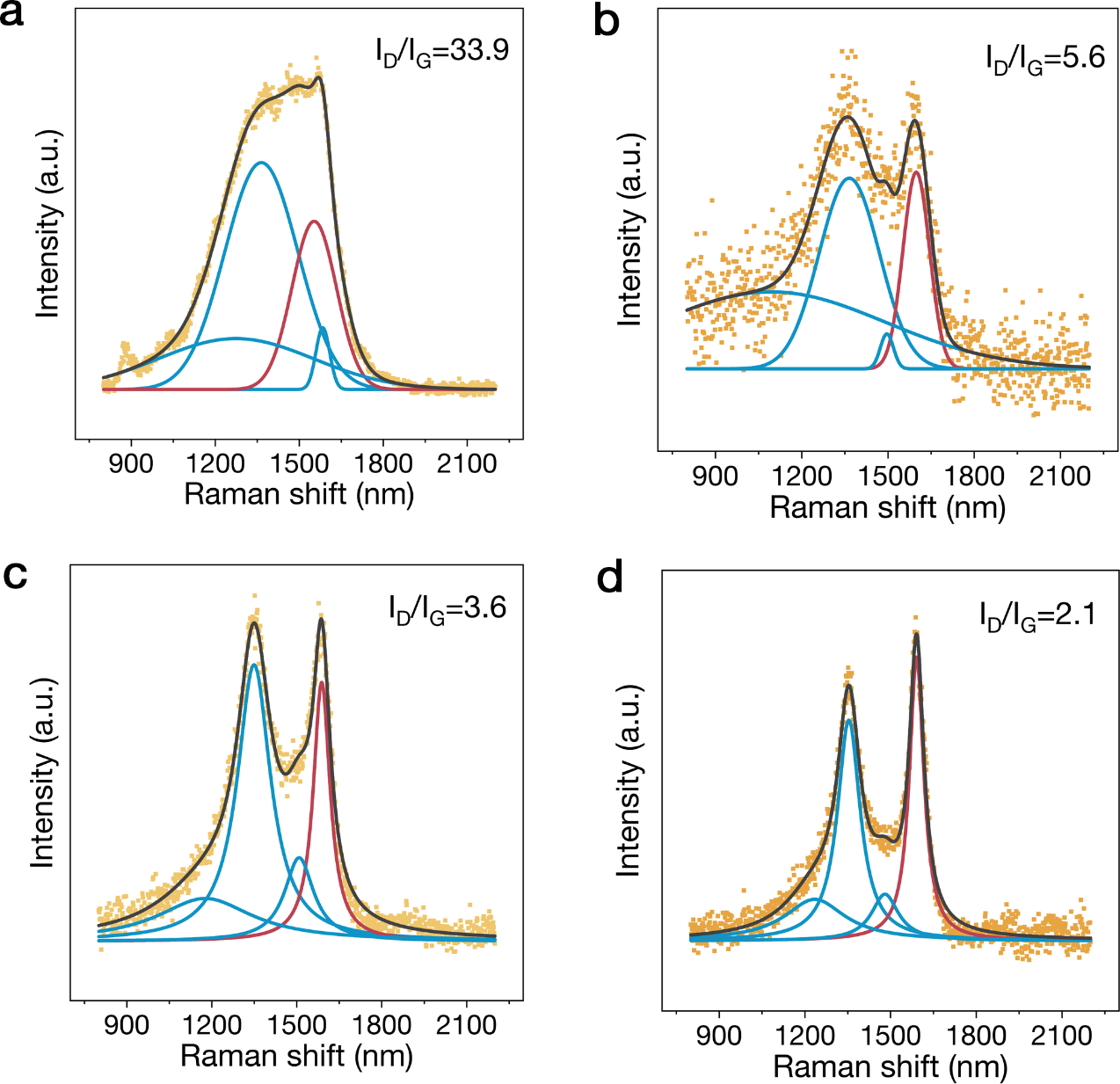


Figure s15. Raman spectra of Co/C-500 (a), Co/C-600 (b), Co/C-700 (c), Co/C-900 (d).

The Raman spectra of Co/C disordered carbon materials typically exhibited two major Raman bands and could be deconvoluted into a G band (~1580 cm^−1^ ), a D3 band (~1500 cm^−1^ ), a D1 band (~1360 cm^−1^ ), and a D4 band (1180 cm^−1^ ), corresponding to graphitic carbon, amorphous sp2 carbon, edge carbon, and sp3 carbon, respectively (Fig. S12). The D band and G band coexisted in all the samples, suggesting that Co/C was composed of graphitic CNTs and supported by amorphous hollow carbon spheres. The I_D_/I_G_ value decreased from 33.9 to 2.1 with the increase in temperature, indicating that the degree of graphitization increased with the temperature (Fig. S15).





Figure s16. XRD patterns of Co/C-500, Co/C-600, Co/C-700, Co/C-900.

By increasing the pyrolysis temperature, the characteristic diffraction peaks simultaneously became sharper and more intense, indicating the improvement in crystallinity of the Co NPs. In addition, the full width at half-maximum of the three peaks evidently decreased, manifesting an increase in the average size of the Co NPs on account of the Scherrer equation. Similarly, the increase in the graphitization of carbon can be seen as the diffraction peak intensity of the typical (002) crystal plane of graphitic carbon increasing versus the temperature (Fig. S16). These results were consistent with the TEM images and Raman spectra.


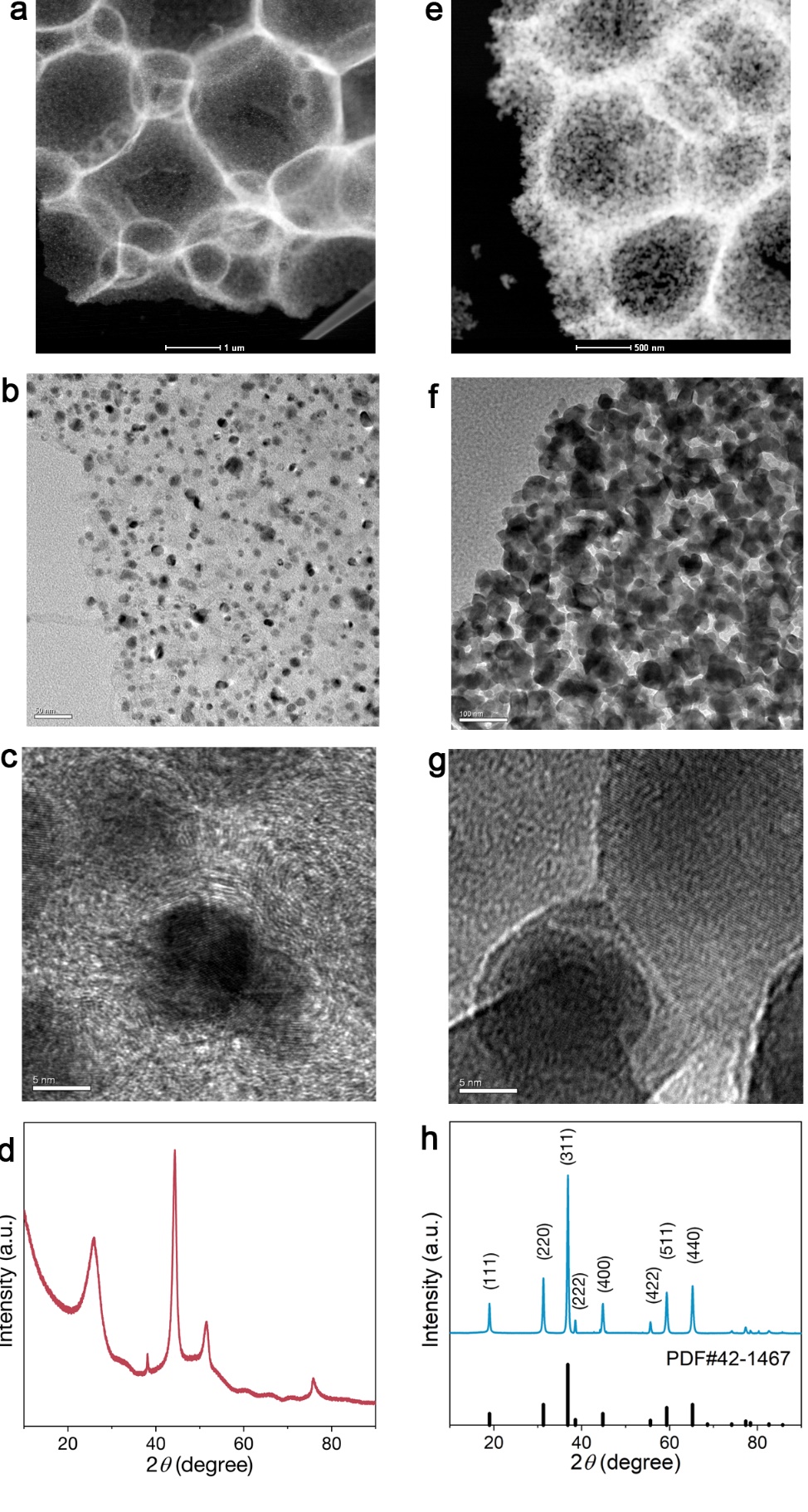


Figure s17. (a-c) STEM and TEM images of Co/C after immersing in HCl (1 M) for 2 hours. (d) XRD pattern of Co/C after immersing in HCl (1 M) for 2 hours. (e-g) STEM and TEM images of Co_3_O_4_/C, which obtained from calcination of Co/C in air at 350 °C for 2 hours. (h) XRD pattern of Co_3_O_4_/C.

The inclusion of carbon can hinder the leaching of the Co NPs. As shown in Fig. S17, after immersing Co/C in hydrochloric acid for 2 hours, a large number of Co NPs were still loaded on the carbon support, and the characteristic diffraction peaks of metallic cobalt were still found in XRD. After calcining cobalt in the air, Co/C was transformed into porous Co_3_O_4_ without obvious changes in morphology, suggesting that the carbon coating had a positive effect in preventing the accumulation of NPs.





Figure s18. The UV-Vis absorption spectra of NADH (100 μM) catalytic oxidation by Co/C in N_2_, air, and O_2_ saturated solutions.


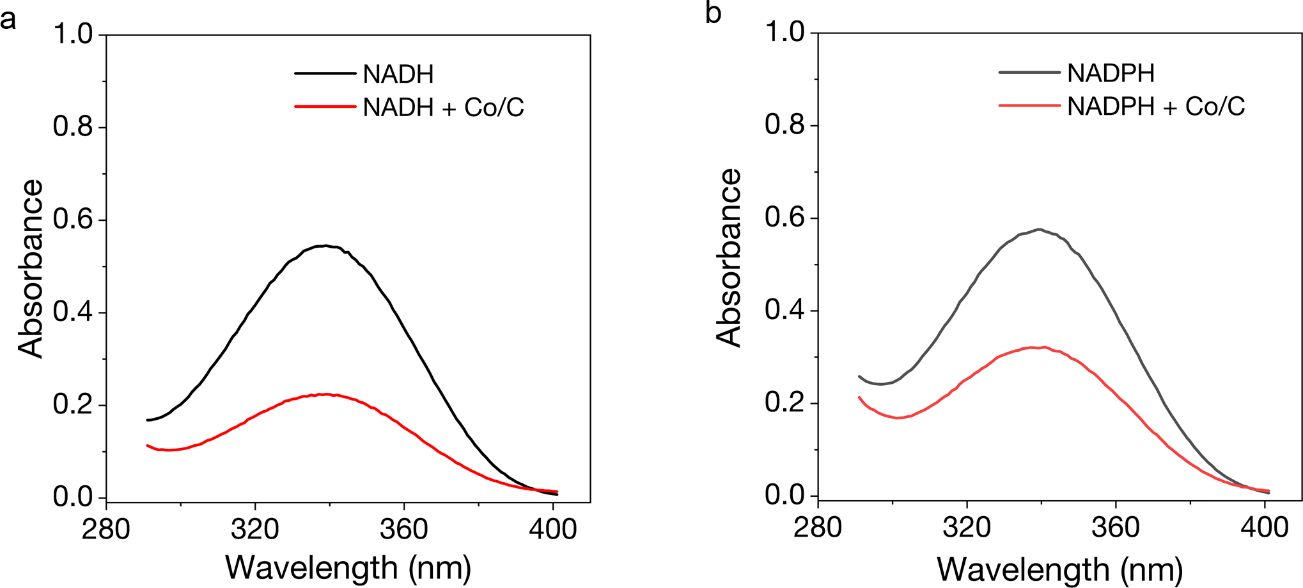


Figure s19. (a) The UV-Vis absorption spectra of NADH oxidation catalyzed by Co/C. (b) The UV-Vis absorption spectra of NADPH oxidation catalyzed by Co/C.





Figure s20. The UV-vis absorption spectra of different mixture after reacting for 1 min.


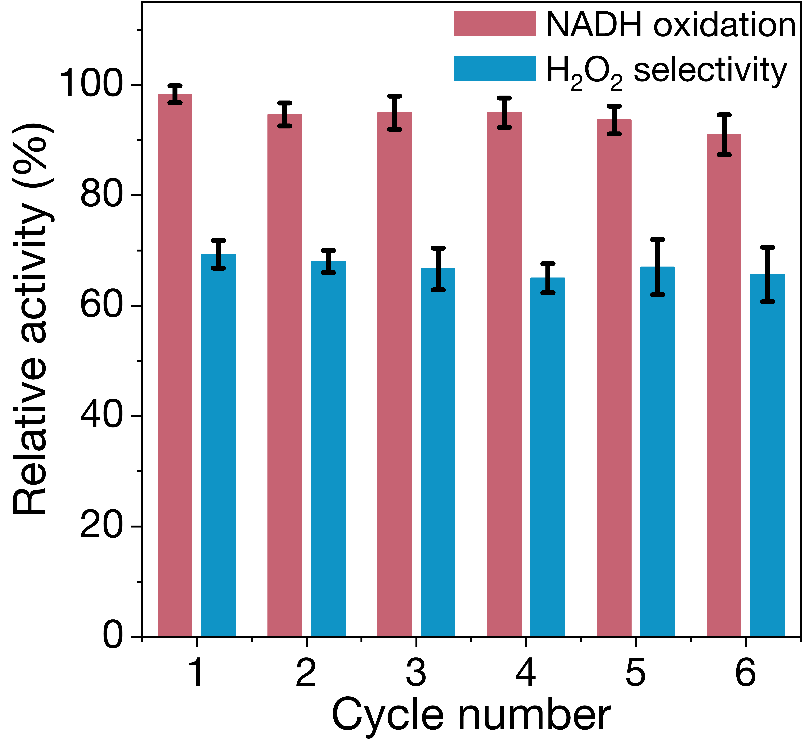


Figure s21. Reusability of Co/C for the NADH oxidation reaction and the corresponding H_2_O_2_ selectivity.





Figure s22. The catalytic activity of Co/C did not decrease obviously after 10 months storage


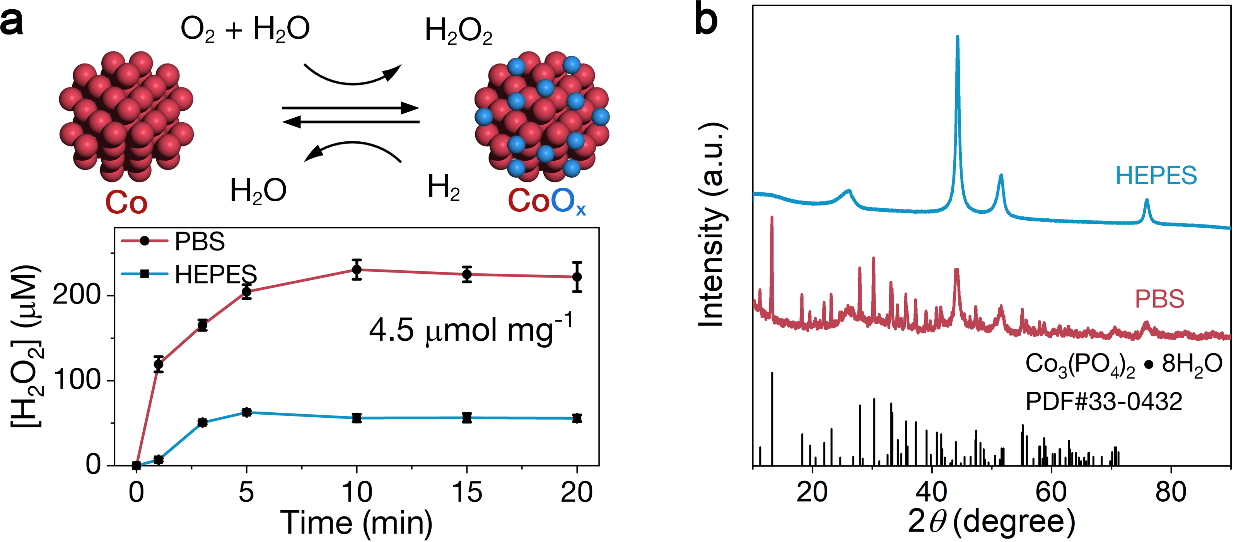


Figure s23. Changes of H_2_O_2_ produced by Co/C (50 μg mL^-1^) in different buffers with time (i) XRD patterns of Co/C after immersing in HEPES (10 mM, pH = 7.4) or PBS (50 mM, pH = 7.4) for 5 hours.


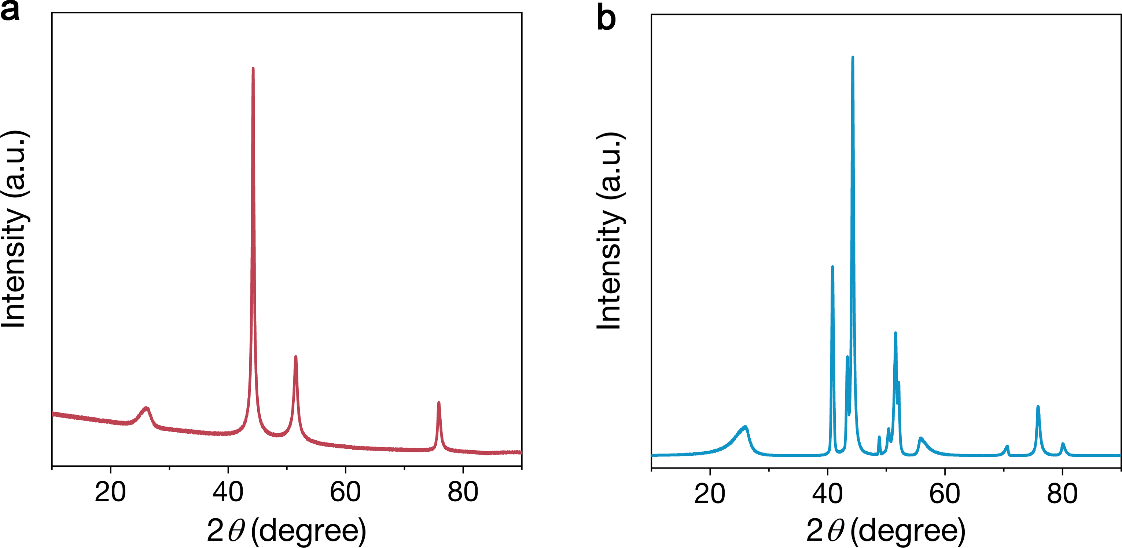


Figure s24. (a) XRD pattern of Co/C after immersing in HEPES (10 mM, pH = 7.4) for 5 hours, then calcined in hydrogen at 800 °C for 2 hours. (b) XRD pattern of Co/C after immersing in PBS (50 mM, pH = 7.4) for 5 hours, then calcined in hydrogen at 800 °C for 2 hours.





Figure s25. (b) The UV-Vis absorption spectra of NADH oxidation in human serum catalyzed by Co/C.


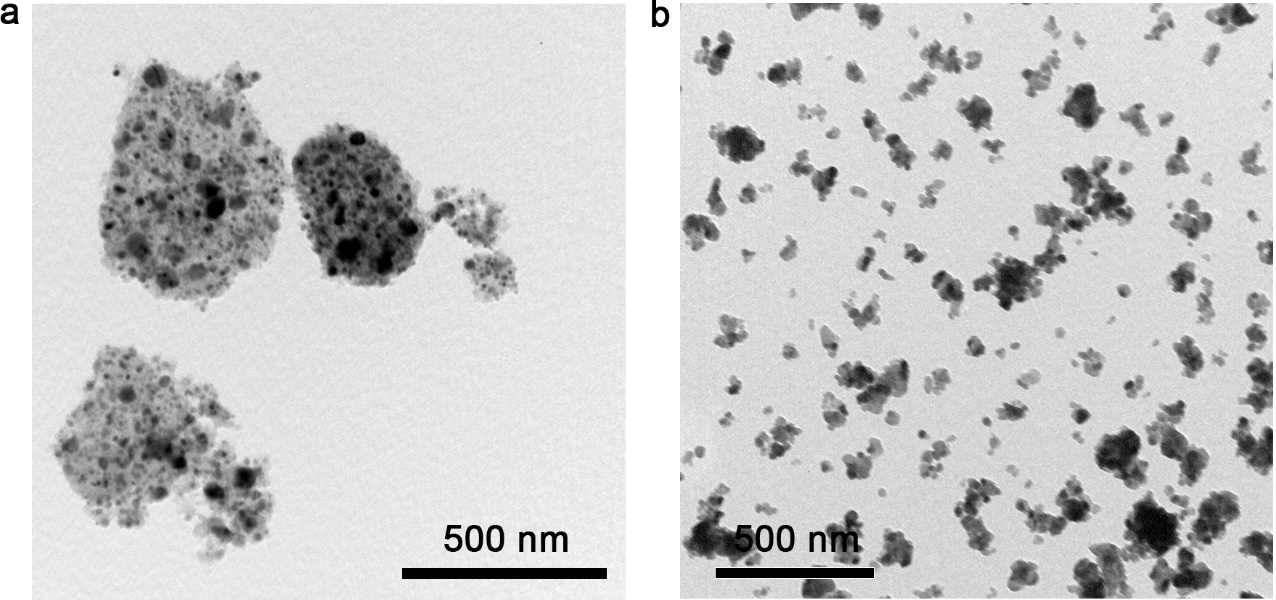


Figure s26. TEM images of Co/C (a) and Co_3_O_4_/C (b) after grind for 20 minutes.


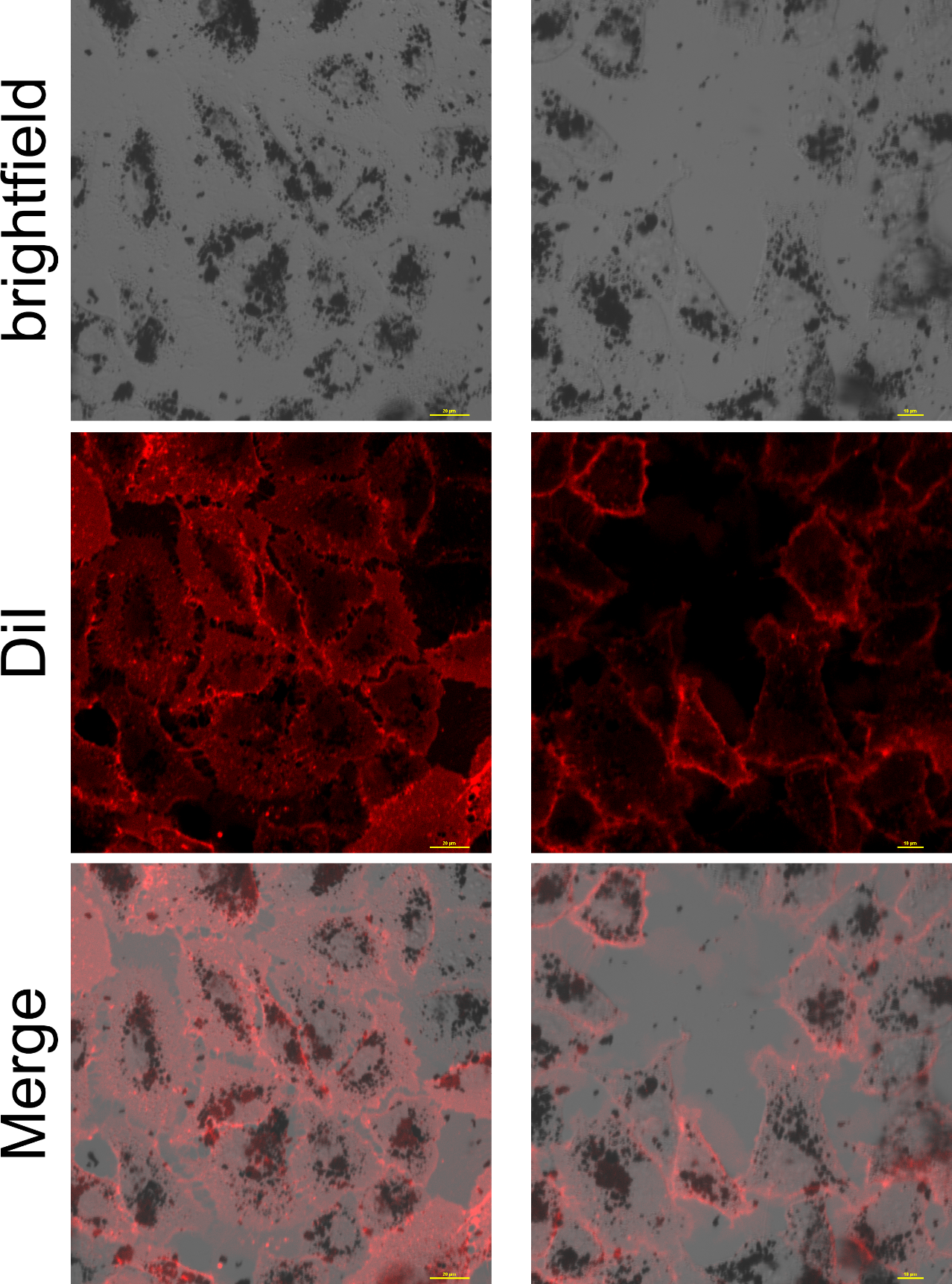


Figure s27. CLSM images of Co/C distribution in cells. Cell membranes was stained by DiI.





Figure s28. HL-7702 cell viability after being treated with Co/C or Co_3_O_4_/C nanoparticles with different concentrations.


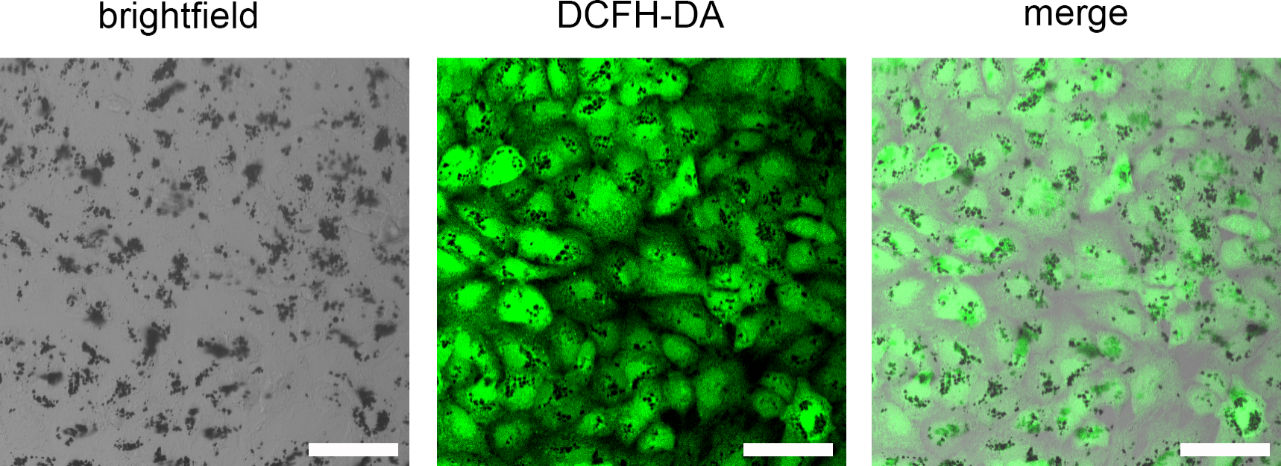


Figure s29. CLSM images of A549 cells stained by DCFH-DA after treated with Co/C nanoparticles for 6 h. The black spot in the fluorescence image is due to the quenching of fluorescence by Co/C. Scale bars: 50 μm.

Table s1. ICP-OES analysis results of the as-synthesized catalysts.

| **Catalysts** | **Co/C-500** | **Co/C-600** | **Co/C-700** | **Co/C-800** | **Co/C-900** |
| --- | --- | --- | --- | --- | --- |
| **Co (wt%)** | 42.3 | 45.6 | 47.1 | 49.6 | 51.3 |

Table s2. The BET surface area, pore volume, and pore size of different catalysts.

| **Catalysts** | **BET surface area** | **Pore volume** | **Pore size** |
| --- | --- | --- | --- |
| **Co/C-500** | 205.2 m^2^/g | 0.232 cm^3^/g | 5.01 nm |
| **Co/C-600** | 230.0 m^2^/g | 0.221 cm^3^/g | 4.82 nm |
| **Co/C-700** | 256.2 m^2^/g | 0.278 cm^3^/g | 4.52 nm |
| **Co/C-800** | 346.4 m^2^/g | 0.303 cm^3^/g | 4.31 nm |
| **Co/C-900** | 334.3 m^2^/g | 0.309 cm^3^/g | 2.00 nm |

Table s3. Comparison of the kinetics of Co/C and natural NOX.

| **Catalysts** | ***K*m (μM)** | **Specific activity (U mg^-1^)** |
| --- | --- | --- |
| **Co/C** | 70.5 | 1.2 |
| **Natural NOX** | 124 | 81.9 |
